# The role of dataset integrity, calibration and signal quality in neural network-based blood pressure estimation

**DOI:** 10.1038/s41598-026-66882-7

**Published:** 2026-08-15

**Authors:** Christian Fritzsche, Veit Senner

**Affiliations:** TUM School of Engineering and Design, Professorship of Sport Equipment and Sport Materials, Technical University of Munich, Munich, Germany

**Keywords:** Photoplethysmography, Blood pressure prediction, Cuffless, Neural network, Cardiology, Computational biology and bioinformatics, Engineering, Health care, Medical research, Physiology

## Abstract

Cuffless blood pressure (BP) estimation via photoplethysmography (PPG) and machine learning has been widely studied, yet reported accuracy remains below clinically acceptable levels, limiting adoption in sport, ambient living, and telemedicine. This study systematically examines potential error sources underlying these performance gaps, with emphasis on: dataset selection, signal synchronization, subject-specific calibration, and data leakage. Three neural networks—a multilayer perceptron (MLP_BP), residual network (Res_BP), and temporal convolutional network (TCN_BP)—were evaluated on two curated public datasets derived from MIMIC III and VitalDB, differing primarily in signal synchronization. MLP_BP uses scalar features from ECG and PPG signals, including pulse arrival time, heart rate, and variability; Res_BP and TCN_BP additionally process PPG signals. Subject-wise splits prevent data leakage, and calibration is applied using three to nine initial reference measurements per subject. Models trained on VitalDB consistently outperform those trained on MIMIC III, particularly for systolic BP, underscoring the importance of synchronized signals. Subject-specific calibration improves performance, with diminishing returns beyond three measurements. Calibrated models reach clinically acceptable accuracy for diastolic BP, while systolic BP remains challenging using PPG alone. These findings suggest that dataset integrity and proper calibration may matter more than model architecture in cuffless BP estimation.

**Supplementary Information:**

The online version contains supplementary material available at 10.1038/s41598-026-66882-7.

## Introduction

Among the major modifiable risk factors, such as cigarette smoking, diabetes mellitus and lipid abnormalities, high blood pressure (BP) is associated with the strongest evidence for the causation of cardiovascular disease (CVD)^1^. Even more problematic is that hypertension remains undetected in 51% of men and 41% of women worldwide. Therefore, the WHO labeled this problem *a race against a silent killer* in the Global Report from 2023^2^.

Conventionally, BP is measured with a sphygmomanometer with an inflatable cuff. However, these measurements are inherently intrusive (a cuff squeezes the arm to block and then release blood flow) and provide only intermittent readings. As a result, a patient’s physiological state may not be adequately captured, and critical signals can go unnoticed since certain risk patterns appear only sporadically or during periods of cardiovascular stress^3^. Although upper-arm and wrist sphygmomanometers are generally affordable and widely accessible, the inflation of the cuff may cause discomfort, which can reduce user compliance. This limitation is particularly relevant for population-wide screening and longitudinal monitoring, where repeated measurements are necessary. Reduced adherence to regular BP assessment contributes to a substantial proportion of undiagnosed hypertension cases.

Moreover, several additional factors can introduce errors in cuff-based blood pressure measurements. These include the number of measurements taken, inappropriate cuff size, improper body positioning, and environmental influences such as masked or white coat hypertension^4^.

Beyond resting BP assessments conducted in clinical or home settings, exercise-induced hypertension is frequently overlooked, despite its established association with masked hypertension and elevated cardiovascular risk. This consideration may be particularly relevant for individuals engaging in regular athletic training^5,6^.

Taken together, these limitations underscore the need for a measurement system capable of frequent, non-intrusive, and comfortable BP monitoring over extended periods. A cuffless approach that enables repeated measurements throughout daily activities, including during and after exercise, could improve the detection of early hypertensive patterns, enhance measurement accuracy, increase user adherence, and facilitate more personalized exercise prescription.

In recent years, many researchers have explored the potential of cuffless blood pressure measurement via single- or multi-sensor approaches, which are typically based on electrocardiography (ECG) or photoplethysmography (PPG). PPG is particularly attractive, as it is a well-established technique used in wearable devices such as fitness trackers and smartwatches to monitor heart rate and heart rate variability. The main objective is to extract features from the measurement signals that are correlated with BP. With the rapid progress in machine learning (ML) and deep learning (DL), along with the availability of several large public datasets, an increasing number of studies have focused on modeling the complex relationships between BP and PPG/ECG signals^7^. Building on this foundation, various methodological approaches have been proposed for cuffless blood pressure estimation, which can be broadly divided into two categories: those that rely solely on PPG signals and those that combine PPG with ECG signals.

PPG-only approaches typically focus on extracting morphological features directly from the PPG waveform. These features may include the amplitude, slope, and timing of characteristic points such as the systolic peak, diastolic peak, and dicrotic notch and characteristic wavepoints (e.g., a/b/c-wave) in the second derivative of the PPG waveform. The characteristic points of PPG and its derivatives are described in^8^. In addition, other studies employ signal decomposition methods—such as semi-classical signal analysis (SCSA)—to extract further features such as eigenvalues and systolic/diastolic invariants^9,10^.

PPG + ECG approaches exploit the complementary information provided by both signals. A widely used feature in this context is the Pulse Arrival Time (PAT), which represents the time delay between the R-peak in the ECG signal and a specific fiducial point in the PPG waveform (e.g. PATp = R-peak to PPG peak). Because PAT and its related measure, Pulse Transit Time (PTT), are influenced by arterial stiffness and other cardiovascular properties, they are often considered potential indicators of blood pressure. However, their relationship with blood pressure is complex and can be affected by multiple physiological and environmental factors^11,12^. Therefore, while timing-based features, such as PAT and PTT, can provide valuable complementary information, they are typically used in combination with morphological features to increase the robustness and accuracy of cuffless blood pressure estimation models.

Regardless of the specific signal-processing strategy, most ML and DL approaches rely heavily on publicly available datasets. Among these, Medical Information Mart for Intensive Care—MIMIC^13^ and its subsets (MIMIC I–IV) are the most widely used, offering a range of physiologic signals—including electrocardiogram (ECG), arterial blood pressure (ABP), and photoplethysmography (PPG)—collected from intensive care unit (ICU) patients.

A number of studies^9,10,14–20^ have demonstrated successful blood-pressure (BP) estimation using PPG signals alone, PPG-derived pulse wave analysis (PWA) features, or combinations of PPG and ECG, along with features computed from them, such as the PAT. Many of these works report performance levels that satisfy the AAMI^21^ and/or BHS^22^ validation standards.

Because machine-learning and deep-learning models learn directly from their training data, both the dataset quality and the preprocessing applied throughout the training pipeline critically influence the resulting model performance. Here, performance denotes the model’s ability to generalize to unseen test data. A model that performs well on the training set but poorly on the test set is likely capturing noise or overly specific patterns in the training data. In some cases, this may involve memorizing training samples rather than learning generalizable patterns, an issue known as *overfitting*. In combination with *data leakage* (data from the same patient appearing in both the training and test sets), this can lead to artificially inflated performance.

For example^17^, presented a model trained on MIMIC-II that predicted BP using only PPG signals, achieving Pearson correlation coefficients of 0.947 and 0.977 for DBP and SBP, respectively. The corresponding MAEs were 2.23 mmHg (SD 2.44 mmHg) for DBP and 3.21 mmHg (SD 3.35 mmHg) for SBP, thereby fulfilling the AAMI requirements and achieving Grade A according to the BHS protocol. However, the authors did not clearly describe how the dataset was partitioned into training, validation, and test sets, opening the possibility of *data leakage* within the training pipeline. Similarly, for^23^, the 150,000 samples of all the subjects were randomly shuffled, leading to patient-level data leakage and, consequently, potentially patient identity memorization rather than true generalization. Another data-processing factor that can artificially inflate model performance is *restricting the dataset* to simplify the prediction task. As an illustration, the final dataset of^20^ did not include patients with DBP ≤ 60 mmHg, although the AAMI standard requires ≥ 5% of patients to have a DBP ≤ 60 mmHg.

The work of^24,25^ demonstrated how typical pitfalls—such as *data leakage* or overly restrictive preprocessing steps (e.g., removing physiologically plausible but atypical samples as “outliers,” thereby simplifying the task)—can inflate the reported accuracy of a model. They demonstrated that the same BP prediction model can appear significantly more accurate when trained on a differently processed version of the dataset, highlighting the sensitivity of BP prediction results to data handling choices.

The dataset structure itself also strongly affects the model performance of any ML or DL model. For example, when patient identifiers are removed, as in BP-UCI^20^, a subset derived from MIMIC II, data leakage becomes unavoidable because patient samples cannot be reliably separated into distinct training and test sets. This also introduces an imbalance, as it becomes impossible to control how much data from any individual patient is displayed during training. These issues directly undermine a model’s ability to generalize and can lead to artificially inflated performance when evaluated on a truly subject-disjoint test set (Table 1).

**Table 1:** Common Pitfalls in ML/DL-based blood pressure prediction.

Pitfall	Mechanism	Outcome
Test Set Bias	Restricting BP distribution	Model tuned on easier subpopulation Hypertensive and hypotensive patients are underrepresented AAMI requirements (population distribution) might be violated
Subject imbalance	Missing sample identifier	Inflated performance driven by a small subset Few Subjects dominate sample count
Data leakage	Subject data appears in both train and test set (e.g. via random split)	Inflated accuracy due to subject-specific memorization

To address these limitations, prior works^26,27^ curated, cleaned subsets of the MIMIC and VitalDB databases. These revised datasets assign unique identifiers to each subject and ensure equal data per patient, while preserving the original raw PPG, ABP, and ECG recordings.

The aim of this study was to train neural network models on these curated datasets^26,27^ and compare their performance with previously reported results. We further investigate how model performance varies across these databases and evaluate the improvements achievable through subject-specific calibration and the inclusion of waveform inputs approximating the actual blood pressure signal.

## Methods

### Datasets

#### MIMIC-BP

The MIMIC-BP dataset from^26^, which is derived from the MIMIC-III Waveform Database^28^, address the issues of class imbalance and patient identification by ensuring that each subject has a unique identifier, that all required signals (PPG, ABP, and ECG) are available, that excessively noisy recordings are removed, and that every patient contributes the same amount of data (1524 subjects with 30 segments of 30 s each). The dataset comprises more than 380 h of ICU waveform data. In this work, we use it for the data-leakage experiment because each segment is 30 s long, unlike the following datasets, which are 10 s long.

#### PulseDB

Reference^27,29^ stated that the PPG, ABP, and ECG from the MIMIC-waveform data may have a changing delay between the different waveforms, therefore not guaranteeing synchronization between the different waveforms. This lack of synchronization imposes limitations on analyses that depend on temporal alignment, including models that incorporate pulse arrival time (PAT) as an input feature. The PAT is defined as the temporal interval between the R-peak of the ECG signal and a reference point on the corresponding PPG waveform (e.g., the systolic peak).

To investigate this limitation, we used two PulseDB^27^ subsets: one derived from VitalDB^30^ with synchronized waveforms (Pulse_Vital) and another from the MIMIC-III Waveform Database (Pulse_MIMIC). In Pulse_Vital, each subject contributes a fixed number of 360 segments for training, whereas Pulse_MIMIC shows wide variability, with subjects contributing anywhere from tens to thousands of segments. To standardize the datasets, we included only subjects with at least 300 segments, and, when more data were available, we selected 300 segments per subject using farthest-point sampling to maximize SBP variability.

Following this selection, Pulse_Vital comprised 1,437 subjects (~ 1450 h of synchronized waveform data) and Pulse_MIMIC comprised 1,516 subjects (~ 1300 h), with signals sampled at 125 Hz. All mentioned datasets are publicly available.

**Fig. 1:**
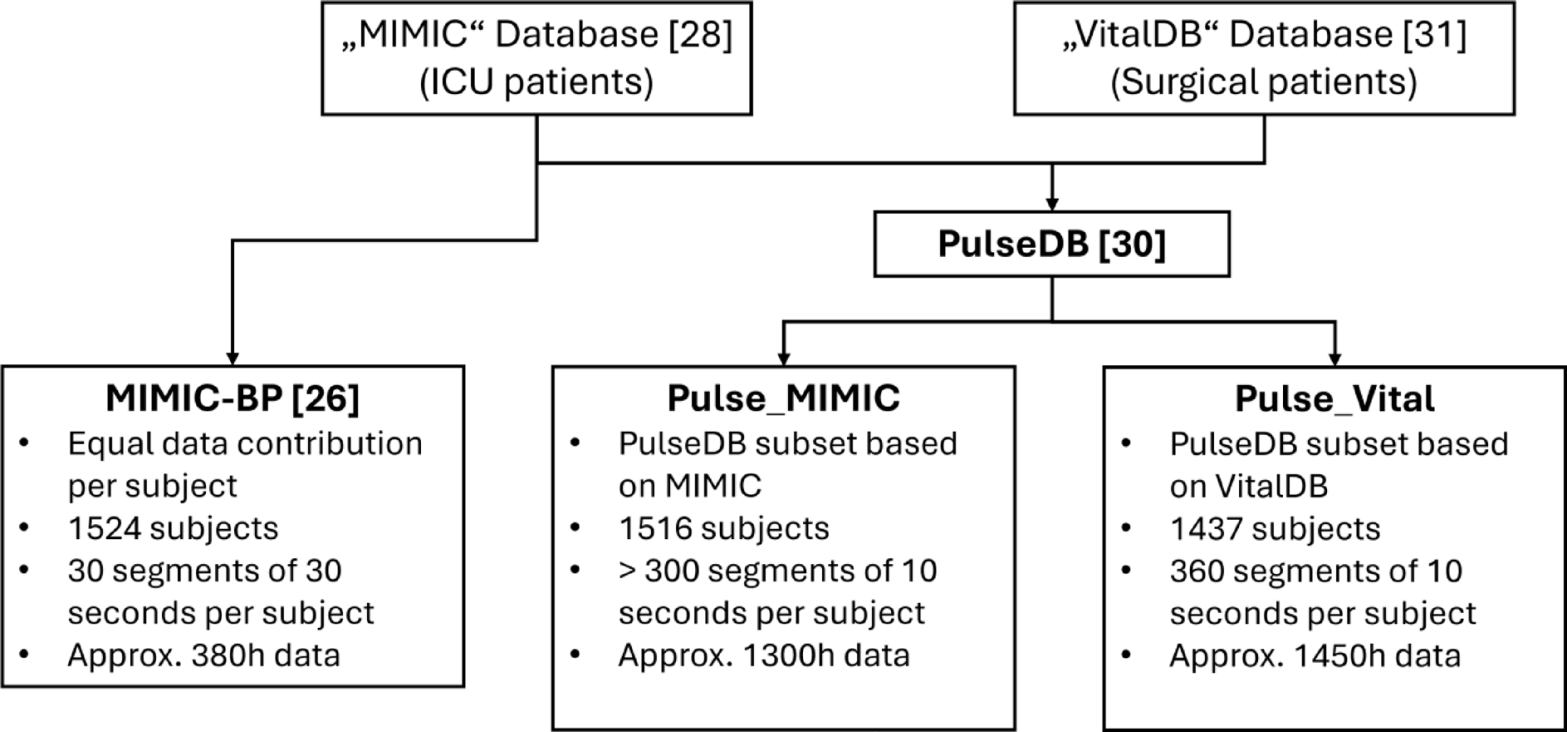
Dataset structure overview

A short overview of the dataset structure and origin is displayed in Fig. 1.

#### Preprocessing

In the preprocessing pipeline, each segment was processed in MATLAB to identify systolic and diastolic peaks in the PPG and ABP signals, as well as R-peaks in the ECG. From each segment, a single 8.5 s window was extracted and resampled to 680 samples, and the remaining portion of the segment was discarded. This approach ensures that each segment contributes only one window to the dataset, thereby minimizing data overlap.

For each detected beat within a window, the features PATf, PATs, PATp, and PATic were computed and subsequently averaged to obtain a single representative value per window. The subscripts f, s and p denote the PPG foot, maximum slope, and systolic peak, respectively, while PATic was derived via the intersection method described in^31^. Additional features were computed as 1/PAT^2^ to approximate the inverse relationship between the PAT and blood pressure.

To ensure reliable peak detection, consistency checks were performed across the PPG, ABP, and ECG signals to verify proper alignment of the systolic and diastolic events. Heart rate (HR) and heart rate variability, quantified via the root mean square of successive differences (HRV RMSSD), were also computed and included as scalar features. In total, the resulting scalar feature vector comprised ten values. Segments exhibiting inconsistencies—typically arising from noise or motion artifacts—were excluded from further analysis.

Prior to feature extraction, the PPG signals were z-normalized. In addition, the first and second derivatives of the PPG waveform were computed for each segment, corresponding to velocity plethysmography (VPG) and acceleration plethysmography (APG), respectively. For each window, the mean diastolic blood pressure (DBP), systolic blood pressure (SBP) and pulse pressure (PP = SBP – DBP) were stored as ground-truth labels.

Samples with blood pressure values outside the predefined physiological ranges (DBP < 50 mmHg or DBP > 120 mmHg; SBP < 80 mmHg or SBP > 180 mmHg) were excluded from the training, validation, and test sets. In a second step, the test set was further filtered to approximate the population distribution specified by the AAMI/ESH/ISO standard by removing samples, primarily those with SBP and DBP values close to the population mean, in order to increase the proportion of samples at the distribution tails. The final test set was adjusted such that, for SBP, at least 5% of participants had values ≤ 100 mmHg, at least 5% had values ≥ 160 mmHg, and at least 20% had values ≥ 140 mmHg; for DBP, at least 5% of participants had values ≤ 60 mmHg, at least 5% had values ≥ 100 mmHg, and at least 20% had values ≥ 85 mmHg. Consequently, the resulting test set does not represent the natural blood pressure distribution of the original cohort, but rather a standardized evaluation population designed to enable clinically comparable performance assessment across blood pressure ranges.

### Neural network (NN) training and performance validation

For the subject-wise split (no data leakage), subject identifiers were randomly allocated to the training, validation, or test sets. All samples belonging to a given subject were assigned exclusively to the corresponding subset.

The authors of the Pulse_Vital subset^27^ already provided a dedicated test set with data from 144 subjects. The remaining subjects were split into training (893 subjects) and validation (385 subjects) sets using a 70/30 ratio. In contrast, the MIMIC_BP and Pulse_MIMIC datasets were partitioned into training (1024 subjects), validation (218 subjects), and test (220 subjects) sets using a 70/15/15 split. Note that the total number of subjects in each dataset is smaller than the one reported in Fig. 1, as some subjects were excluded due to filtering criteria (min/max BP) and/or unsuccessful signal segmentation caused by noisy PPG and/or ECG recordings. For model comparison, all models were evaluated on the same test set to ensure consistency. However, for the comparison of training datasets, each model was evaluated on the test set corresponding to the dataset used for training. For example, a model trained on Pulse_Vital was tested on the Pulse_Vital test set, but not on the Pulse_MIMIC test set.

All model training pipelines and neural network architectures were implemented in Python 3.12.3 using TensorFlow 2.20 (CUDA 12.8.1) and Keras. The models were trained using the AdamW optimizer with a learning rate of 7e-5, weight decay of 1e-4, and batch size 32. Training was terminated after 350 epochs or earlier if the validation loss did not improve for 15 consecutive epochs (early stopping). The architecture of all the evaluated models is provided in the Supplementary Information. All experiments were run with a fixed random seed of 42 to ensure reproducibility.

The loss function was defined as the mean absolute error (MAE) computed jointly across the SBP, DBP, and PP output heads. Within the datasets, PAT exhibited the strongest correlation with PP (see the Results section). Therefore, PP prediction was included as an auxiliary task to aid DBP and SBP estimation but was not considered in the quantitative evaluation.

Model performance was evaluated by applying the test set to the trained models and comparing the predicted and reference DBP and SBP values. An overview of all hyperparameters is provided in Table 2.

**Table 2:** Hyperparameter overview.

Parameter	Value
Input Dimension (signal branch)	680 × 3
Input Dimension (scalar branch)	10 × 1
Weight decay	1e-4
Learning Rate	7e-5
Learning Rate Schedule	Linear Warmup during first epoch, held constant afterward
Random seed	42
Batch size	32
Max Epochs	350
Early stopping	15 epochs
Optimizer	AdamW (default settings)
Gradient Clipping	clipnorm = 1.0
Loss function	MAE (Mean Absolute Error)
Framework Version	TensorFlow 2.20, CUDA 12.8.1, cuDNN 9
Precision	Mixed precision: float32
JIT compile (XLA)	True
GPU	NVIDIA GeForce RTX 5070 Ti

### Model architecture

#### MLP_BP

The MLP_BP model is a multilayer perceptron that processes ten scalar input features through a fully connected layer with 16 neurons and ReLU activation, followed by batch normalization and dropout of 0.01. The resulting feature representation is then passed to a 32-neuron dense layer, yielding a shared latent representation for blood pressure estimation. From this shared representation, separate prediction heads for DBP, SBP, and PP are derived, enabling each head to learn task-specific features while exploiting the inherent interdependence among these variables.

#### Res_BP and TCN_BP

The Res_BP and TCN_BP architectures build upon the MLP_BP framework by integrating multichannel temporal signals (PPG, VPG, APG) alongside the scalar input features. The signals are processed in parallel with the scalar features through either a residual network (ResNet) or a temporal convolutional network (TCN), capturing temporal dependencies and multiscale patterns. The outputs of the signal branch are concatenated with the scalar branch and passed through a 32-unit dense fusion layer, producing a shared intermediate representation from which separate DBP, SBP, and PP prediction heads are derived. When calibration data is used, an additional processing branch encodes the calibration signals and scalar features before fusion in the final dense layer.

Both architectures utilize a shared convolutional stem featuring a 1D convolutional layer with 32 filters and a kernel size of 15. Given that input signals are resampled to 680 points over 8.5-s windows, this configuration yields an initial temporal receptive field of approximately 187 ms, sufficient for capturing critical PPG morphological features, including the systolic peak and dicrotic notch. In the Res_BP variant, kernel sizes decrease from 9 to 7 across two residual blocks to establish a coarse-to-fine receptive field. Conversely, TCN_BP employs a series of four blocks with dilation rates of 1, 2, 4, and 8, ensuring the network covers the full 680-sample input while maintaining parameter efficiency. Regularization strategies differ between the two: TCN_BP integrates Batch Normalization, whereas Res_BP uses Batch Normalization in addition to a conservative dropout rate of 0.05. This low dropout rate was selected because no significant overfitting was observed, and higher rates did not yield further improvements in generalization. To further mitigate overfitting risks, we intentionally constrained model capacity by maintaining low filter counts (16–32) and minimizing network depth, thereby forcing the models to learn more robust, generalizable representations of the input signals and subject-specific features.

### Calibration data

Each subject exhibits an individual PAT that reflects their subject-specific blood pressure characteristics. To account for this inter-individual variability, subject-level calibration data are incorporated into the training pipeline for the calibration experiments. For each subject, the first three, six, or nine measurements (3 cal, 6 cal, 9 cal) segments are selected and isolated from the train, val, and test sets, and their corresponding inputs and labels are designated as calibration data. This calibration data serves as an additional conditioning input, providing the model with a baseline representation of subject-specific scalar features (e.g., PAT, HRV) and signal morphology (PPG), along with the associated DBP, SBP, and PP. By providing this individualized baseline information, the model can better adapt to each subject’s physiological profile, thereby improving prediction performance.

The calibration data is processed through a dedicated branch that mirrors the main signal encoder: each calibration PPG segment is passed through a Conv1D stem followed by a ResNet or TCN block, similar to the input signal processing blocks in Res_BP and TCN_BP. These embeddings are then concatenated with the corresponding subject-specific scalar features and associated labels DBP, SBP, and PP. This context vector is then concatenated with the main signal representation and the scalar feature embedding before the final DBP and SBP predictions are made.

**Fig. 2:**
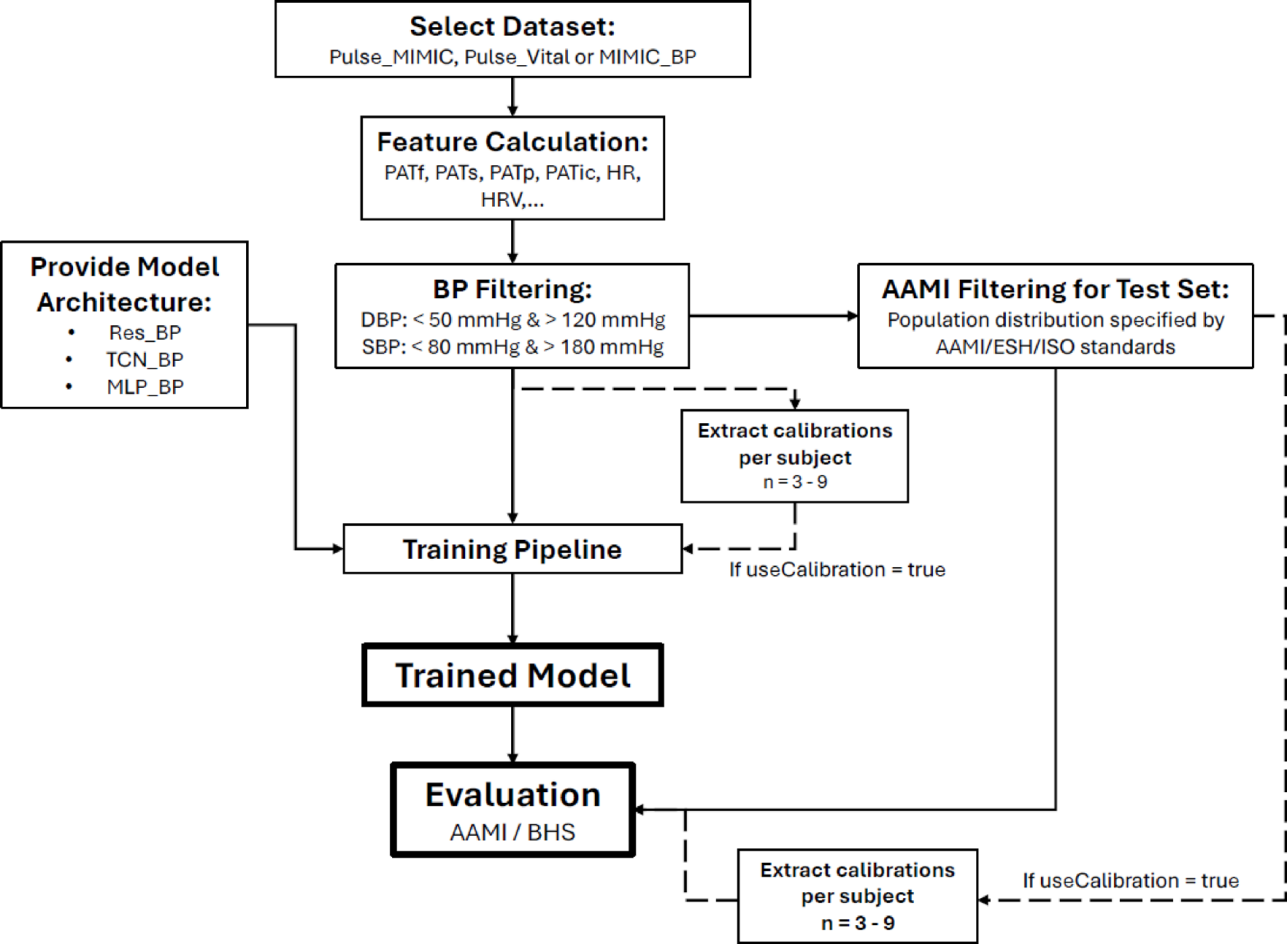
Model training overview

**Fig. 3:**
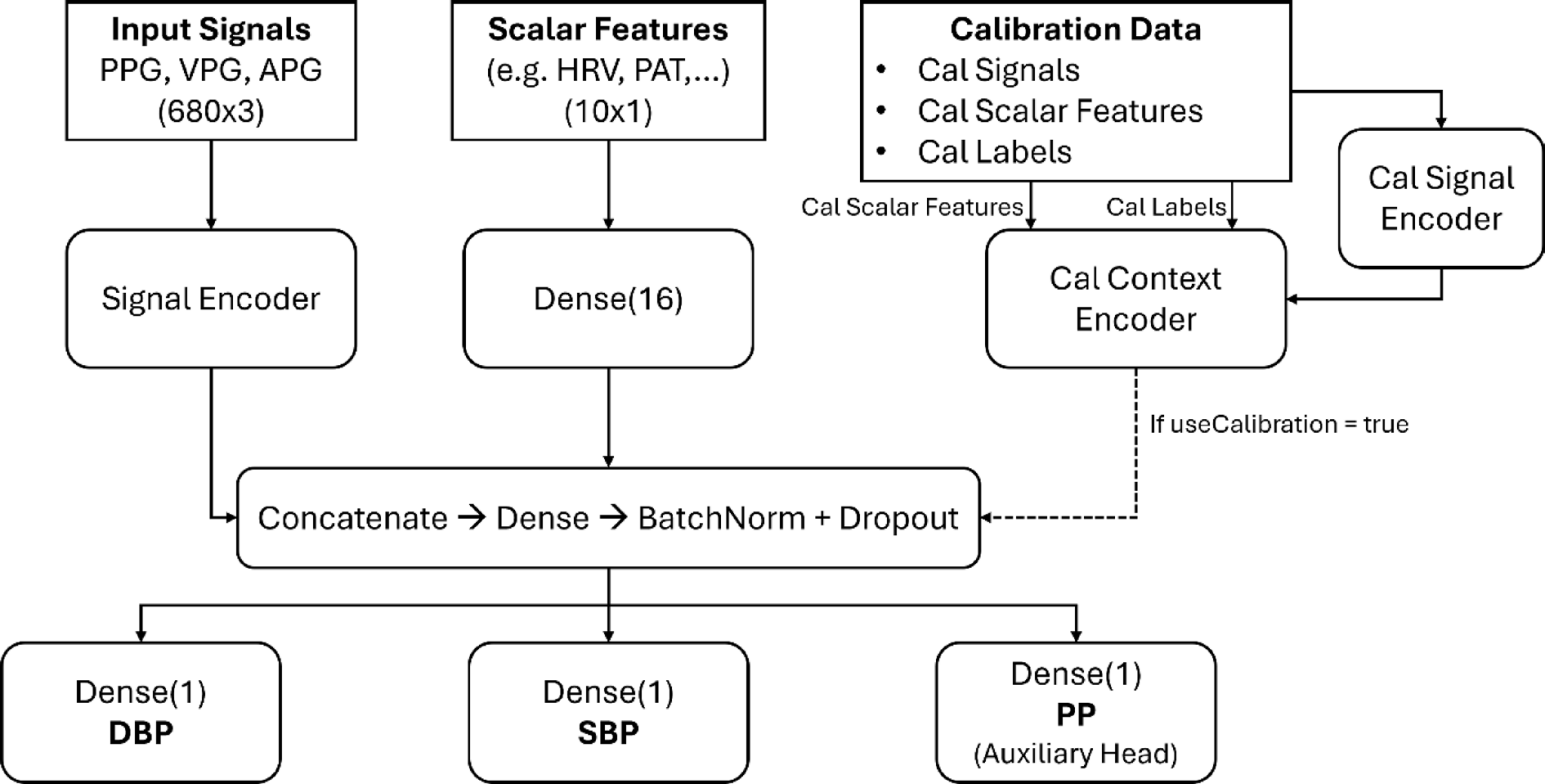
Model architecture overview

Detailed model architectures are provided in the Supplementary Information. A short overview of the training pipeline and model architecture is displayed in Figs. 2 and 3.

### Model evaluation

For individual model performance evaluation, predicted and reference values for DBP and SBP were assessed using multiple metrics. Specifically, the mean error (ME) with its standard deviation (SD), the mean absolute error (MAE) with its standard deviation (STD), and the Pearson correlation coefficient were computed. In addition, the AAMI standard^21^ was assessed, which requires an ME within ± 5 mmHg and an SD not exceeding 8 mmHg. Furthermore, accuracy was evaluated according to the BHS protocol^32^, which assigns grades from A to D, with Grade A indicating the highest level of accuracy.

Statistical comparison of the models was performed on a shared test set, enabling paired analysis. For each model pair (TCN_BP vs. Res_BP, TCN vs. MLP_BP, Res_BP vs. MLP_BP), per-sample absolute errors were computed, and paired differences were tested for normality using the Shapiro–Wilk test. Because the differences were non-normally distributed, the Wilcoxon signed-rank test was used for all comparisons. To control the familywise error rate across six simultaneous tests (3 pairs × 2 outcomes), p-values were adjusted using the Holm-Bonferroni method. Effect sizes were reported as rank-biserial correlations (r). Bootstrap 95% confidence intervals (5000 resamples) were computed on the mean paired difference in MAE.

## Results

### Comparison of Res_BP, TCN_BP, and MLP_BP

Tables 3 and 4 summarize the performance of all models for DBP and SBP when trained on datasets without calibration data and with increasing numbers of subject-specific calibration measurements. For this comparison, we trained and evaluated the models with Pulse_Vital.

**Table 3:**
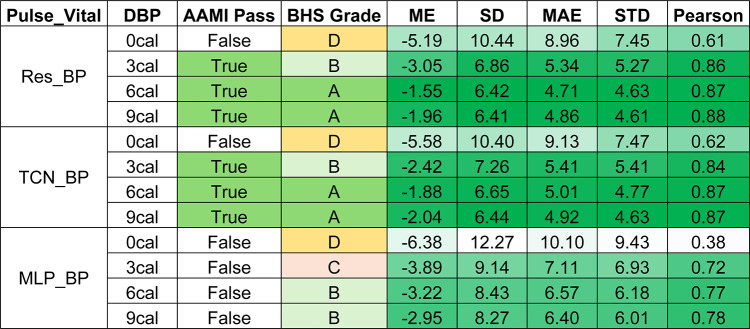
Metrics for the DBP.

**Table 4:**
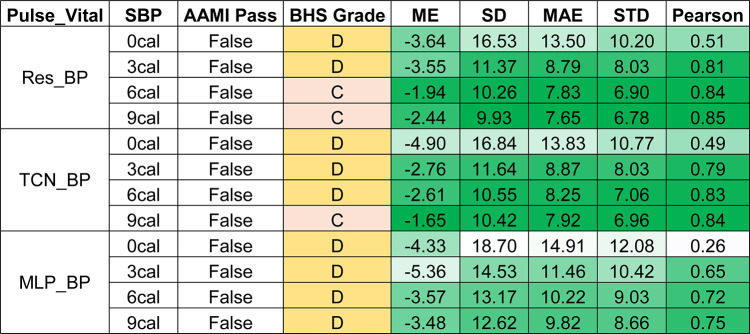
Metrics for the SBP.

Across all architectures, SBP prediction is consistently more challenging than DBP prediction, a trend observed in prior work^9,10,14–16,18–20^.

**Fig. 4:**
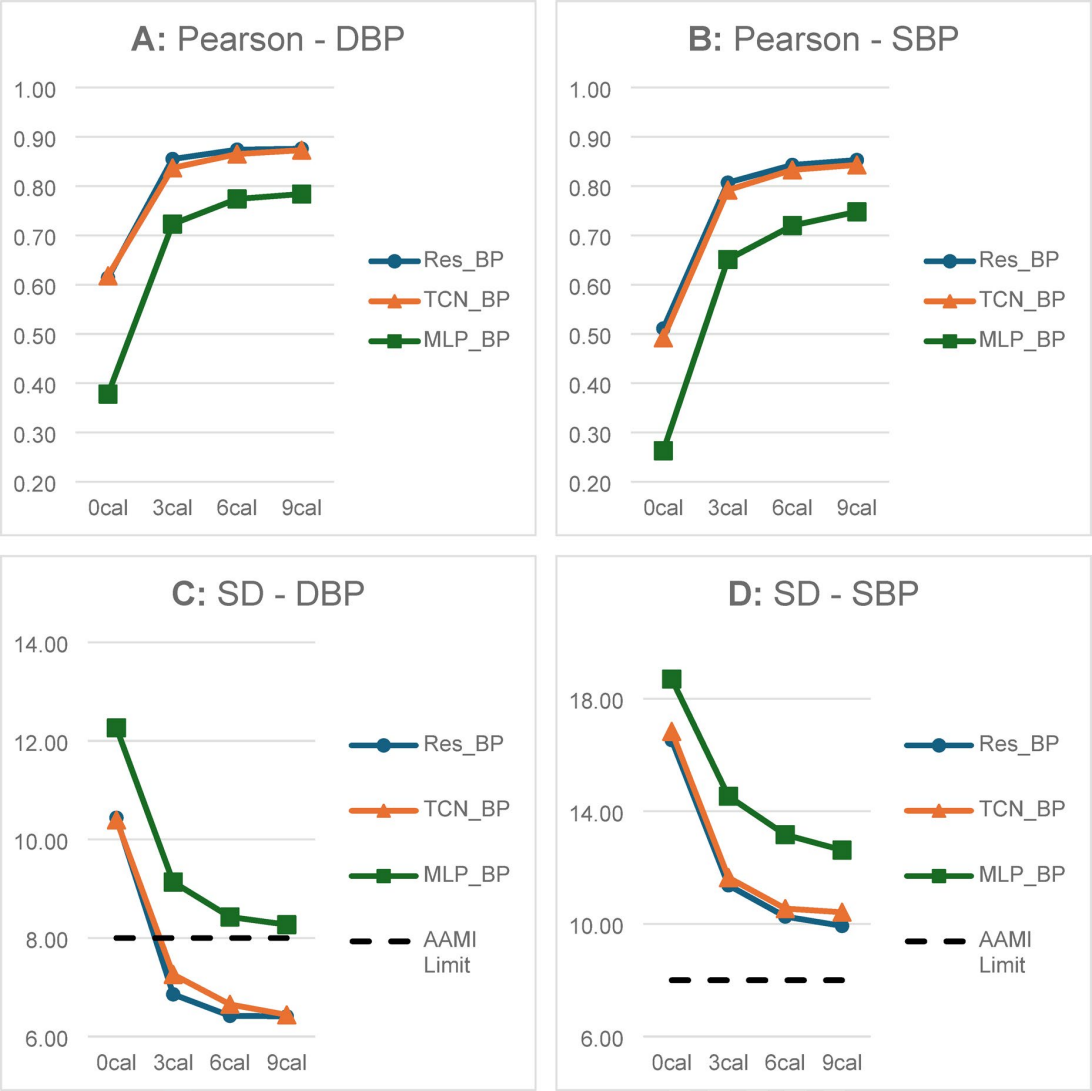
Pearson correlation coefficient and SDs over different numbers of calibrations

Introducing as few as three calibration measurements results in a substantial improvement in performance, as evidenced by higher Pearson correlation coefficients and lower SDs. However, further increases in the number of calibration measurements yield diminishing returns, with performance approaching a plateau (see Fig. 4).

Despite these gains, none of the models meet the AAMI criterion for SBP estimation (SD < 8 mmHg), even when calibration data are included. For DBP estimation, both Res_BP and TCN_BP already meet the AAMI standard after three calibration measurements, whereas the MLP_BP model fails to satisfy this criterion under all calibration conditions.

Regarding model architecture, all pairwise comparisons remained statistically significant after correction for multiple comparisons (all *p* < 0.05). TCN_BP and Res_BP demonstrated comparable performance, with statistically significant yet practically negligible differences in MAE and only small effect sizes (*r* < 0.15). For example, in the three-calibration setting, the comparison between TCN_BP and Res_BP yielded

$$ \begin{aligned} & {\mathrm{DBP}}:\Delta {\mathrm{MAE}} = 0.0{6}\;{\mathrm{mmHg}},\;{95}\% \;{\text{CI }}\left[ { - 0.0{6}, \, 0.{18}} \right],r = 0.0{1}; \\ & {\mathrm{SBP}}:\Delta {\mathrm{MAE}} = 0.0{8}\;{\mathrm{mmHg}},\;{95}\% \;{\text{CI }}\left[ { - 0.{11}, \, 0.{26}} \right],r = 0.0{2}. \\ \end{aligned} $$

In contrast, both TCN_BP and Res_BP substantially outperformed MLP_BP in all calibration conditions, with effect sizes ranging from *r* = 0.14 to 0.45. Exemplarily, in the three-calibration setting, the comparison between Res_BP and MLP_BP showed

$$ \begin{aligned} & {\mathrm{DBP}}:\Delta {\mathrm{MAE}} = - {1}.{76}\;{\mathrm{mmHg}},\;{95}\% \;{\mathrm{CI}}\;\left[ { - {1}.{99},\; - {1}.{55}} \right],r = \, 0.{42}; \\ & {\mathrm{SBP}}:\Delta {\mathrm{MAE}} = - {2}.{66}\;{\mathrm{mmHg}},\;{95}\% \;{\mathrm{CI}}\;\left[ { - {3}.0{5},\; - {2}.{28}} \right],r = \, 0.{36}. \\ \end{aligned} $$

Full results are reported in Supplementary Data.

### Comparison of datasets based on MIMIC or VitalDB

In this section, we retrain the models on the Pulse_MIMIC dataset (based on MIMIC-III^28^) set using either no calibration or three calibration measurements, aiming to determine whether potential waveform asynchronicity in the dataset leads to performance degradation (Table 5).

**Table 5:**
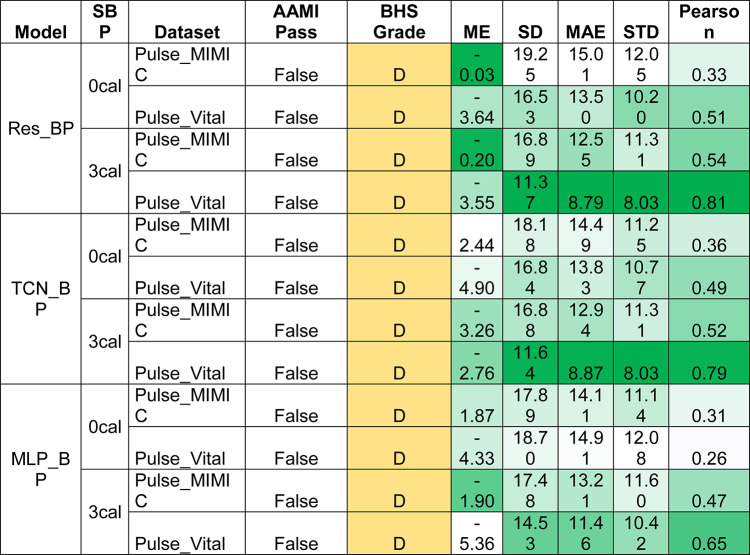
Comparison of the SBP Metrics of models trained with Pulse_MIMIC and Pulse_Vital.

Across nearly all the experiments, the models trained on the Pulse_Vital dataset (based on VitalDB^30^) consistently outperformed those trained on the Pulse_MIMIC dataset, except for MLP_BP (0 cal). We attribute this performance disparity to a limitation previously reported in the literature^27,29^, namely the presence of variable inter-signal delays among the PPG, ABP, and ECG waveforms in the MIMIC-III database. Such temporal misalignment may compromise accurate signal synchronization and thereby reduce the reliability of derived scalar features, particularly PAT.

**Fig. 5:**
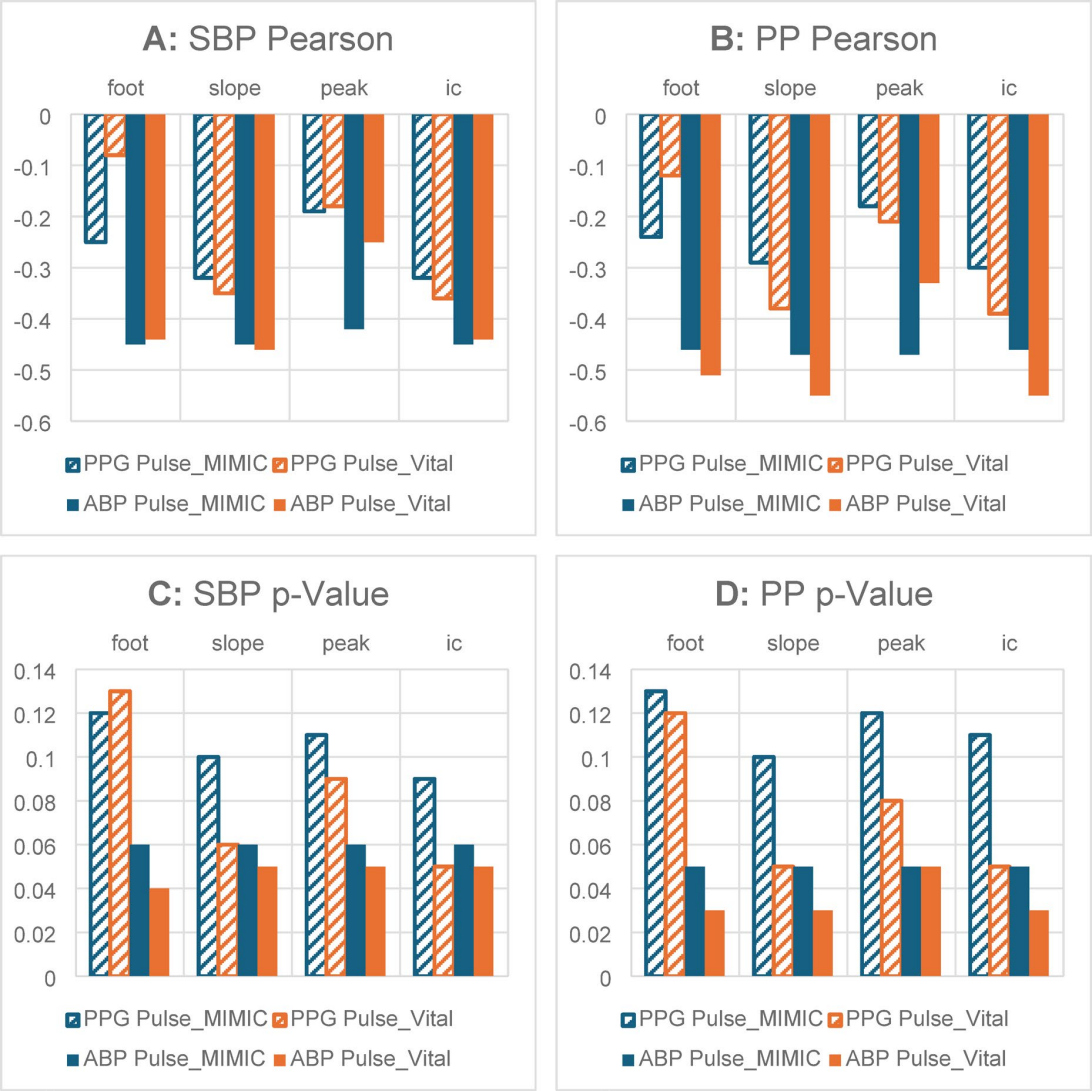
Pearson correlation coefficients for SBP (**A**) and PP (**B**), and corresponding p-values for SBP (**C**) and PP (**D**), evaluated on the Pulse_MIMIC and Pulse_Vital datasets via PPG or ABP signals.

To further examine this issue, we computed the PAT at all corresponding fiducial points between the PPG and ABP signals for both the Pulse_Vital and Pulse_MIMIC datasets (Fig. 5). When PAT was derived from PPG signals, the strongest correlations with systolic blood pressure (SBP) were observed for the PATs and PATic features, with consistently higher correlation coefficients for PP across all fiducial points. These associations were more pronounced in the Pulse_Vital dataset than in the Pulse_MIMIC dataset, as indicated by slightly stronger negative correlation coefficients and lower p-values. In contrast, when PAT was derived from ABP signals, the inter dataset discrepancy was substantially reduced. ABP-derived PAT demonstrated stronger, more statistically significant correlations and lower variability.

To put our calculated correlations into perspective, PAT-SBP correlations have been reported to range from (− 0.37, − 0.54)^33–36^ to < − 0.8^11^. Ranges vary depending on the methods used to detect the PAT, the datasets used, or the condition/task of the subjects. Additionally, in the abovementioned works, the correlation between PAT-SBP is usually greater than that between PAT-DBP, and this is also the case for our subsets (see Supplementary Data). The PAT-PP correlations were even greater (with lower p-values) than the PAT-SBP correlations were. To utilize that, we used the PP as an auxiliary prediction head in all our models, as described above in the Methods section.

We also observed that the correlations varied depending on the method used to calculate the PAT (foot vs slope vs intersect vs peak), with the PATp performing worse and PATic and PATs best in the Pulse_Vital set, aligning with other reported observations^11,35^. One possible reason for this finding^11,31^ is that at any point in the arterial tree, the pressure wave can be considered a summation of forward- and backward-traveling waves, which affects the peak of the PPG signal most.

The observed performance improvement can be largely attributed to the guaranteed temporal synchronization of the PPG, ABP, and ECG channels in VitalDB^30^, which enables more precise estimation of PAT. In contrast, the MIMIC-III dataset^28^ likely contains non-constant inter-channel delays that reduce the reliability of PAT-derived features. Although a fixed temporal offset could, in principle, be learned and compensated for by the model, time-varying delays introduce irreducible noise that cannot be consistently corrected, thereby degrading performance.

It is also important to consider differences in patient populations between the two datasets. VitalDB^30^ comprises surgical patients, whereas MIMIC-III^28^ includes ICU patients. The lower PAT–SBP correlation observed in the Pulse_MIMIC subset may therefore also reflect differences in physiological state and medication exposure, as ICU patients often exhibit greater hemodynamic instability and pharmacological intervention, both of which can alter vascular tone and pulse wave propagation characteristics.

### Influence of auxiliary PP head on DBP and SBP prediction

In the previous section, we observed that PP showed an even stronger correlation with PAT than SBP. Since PATf, PATs, PATp, and PATic were included in the input feature vector, we hypothesized that incorporating PP labels as an auxiliary prediction task could further improve the prediction accuracy of DBP and SBP.

**Fig. 6:**
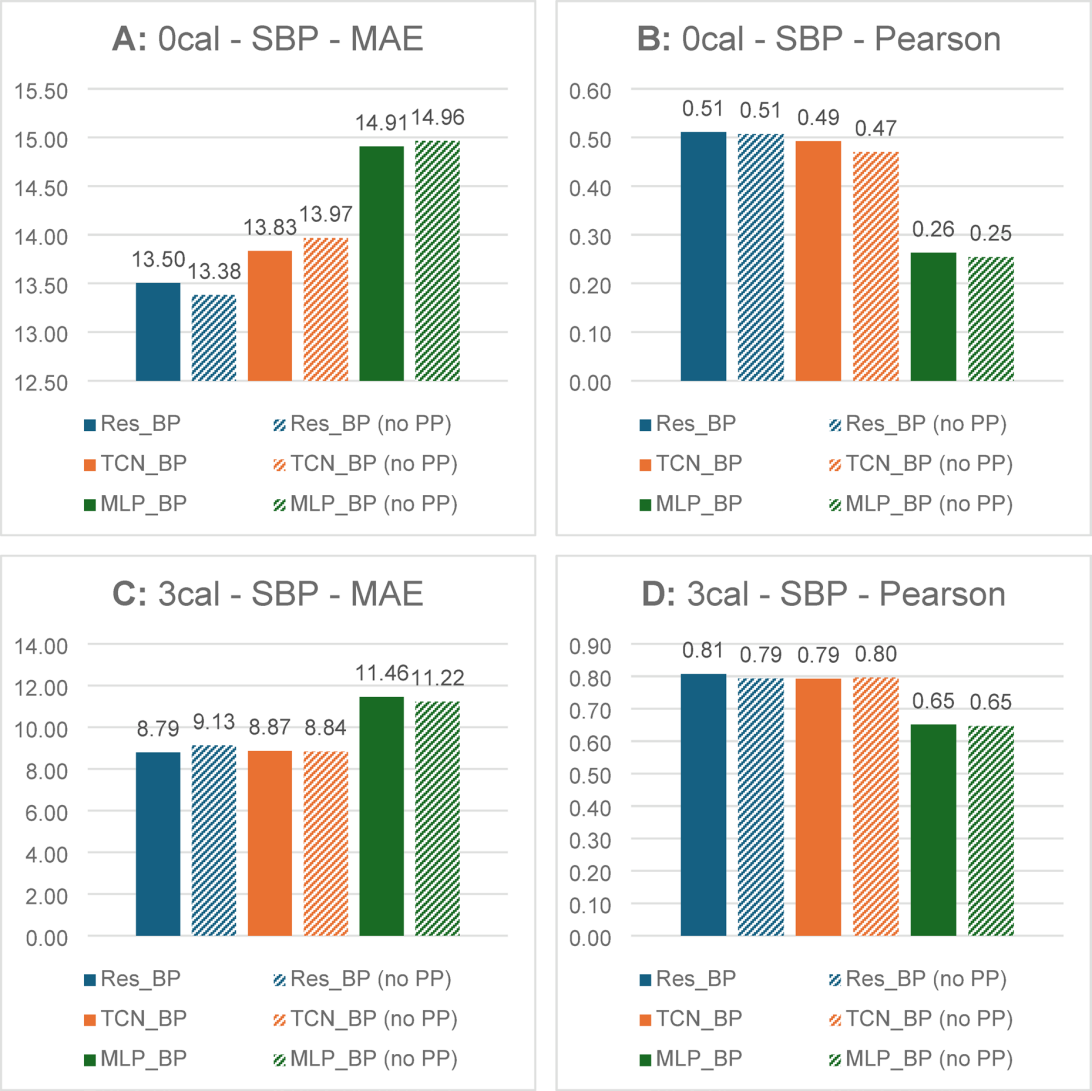
Comparison of Model Performances for SBP Prediction with and without (no PP) auxiliary PP head

To evaluate this hypothesis, we compared the MAE and Pearson correlation coefficients for DBP and SBP predictions from models trained with and without the auxiliary PP prediction head, with no calibration and three calibrations (Fig. 6). For clarity, only SBP results are presented in this section, as SBP prediction represents the more challenging task. The corresponding DBP metrics are provided in Supplementary Data.

Overall, models with and without the auxiliary PP head performed comparably. For instance, comparing Res_BP with Res_BP (no PP) under the no-calibration condition yielded only a marginal difference (ΔMAE = 0.12 mmHg, 95% CI [− 0.09, 0.34], r = 0.028). Slightly larger differences were observed under the three-calibration condition (ΔMAE =  − 0.34 mmHg, 95% CI [− 0.49, − 0.18], r = 0.114), though the overall effect size remained small. Similarly, comparing TCN_BP with TCN_BP (no PP) revealed negligible differences (ΔMAE = 0.03 mmHg, 95% CI [− 0.15, 0.21], r = 0.001).

One plausible explanation for the limited impact of the auxiliary PP head is that PP is not an independent physiological quantity but is directly derived from SBP and DBP (PP = SBP − DBP). Since the network already jointly predicts SBP and DBP from a shared latent representation, the auxiliary PP task likely provides only a marginal additional supervisory signal during optimization. As a result, the auxiliary task may have acted as a redundant constraint rather than introducing complementary physiological information capable of substantially improving blood pressure estimation performance.

### Comparison with other models

Some studies have reported high predictive performance, with correlation coefficients ranging from 0.966 to 0.977 for systolic blood pressure (SBP) and from 0.947 to 0.964 for diastolic blood pressure (DBP), along with standard deviations (SD) of 3.35 mmHg and 2.44 mmHg^17,37^, passing the AAMI test and receiving a BHS Grade A for DBP and SBP. Both models were trained on MIMIC-based datasets.

Notably, the AAMI test requires the standard deviation of the mean error (ME), not of the mean absolute error (MAE), which was calculated in the above-mentioned studies. Usually, the SD of the ME is bigger than that of the MAE because neglecting the sign of the error reduces the variance^17,37^.

In this work, we wanted to investigate the extent to which data leakage may influence evaluation metrics. Therefore, we re-examined the models described in^17,37^ because they were well documented. In addition, we added reduced-capacity variants of these models by reducing the layer depth and number of filters in the DeepBP^37^ model (denoted as DeepBP small), and the hidden layer width and depth in the MLP OG^17^ model (denoted as MLP OG small). These reduced-capacity variants were included to assess how model capacity affects data leakage and evaluation performance.

In the first experiment, the models were retrained using the MIMIC-BP dataset, which applies a subject-wise data split while maintaining an equal number of samples per subject. Furthermore, instead of extracting a single 8.5 s window from each segment, we generated multiple non-overlapping 2 s windows per segment, a common strategy for increasing dataset size. The MIMIC-BP dataset was selected over Pulse_MIMIC because its 30 s segments, compared with the 10 s segments in Pulse_MIMIC, allowed approximately three times as many samples to be extracted from each segment. This characteristic is particularly relevant for investigating potential data leakage effects. When multiple short windows are generated from the same underlying physiological segment, the resulting samples are inherently highly correlated, despite being non-overlapping. Consequently, if leakage exists between training and test partitions, the larger number of derived windows in MIMIC-BP is expected to amplify its impact on evaluation metrics. In the following section, we report the metrics for SBP, as it’s generally more challenging to predict than DBP.

Using our dataset and the subject-wise data split in our training pipeline, we were unable to reproduce the reported high correlations and low MAEs. In fact, our models showed lower errors and higher Pearson correlations. Overall, the MEs and SDs were in the range of previously reported models, ResNet152^26^ and PPG2ABP v2^38^, where a subject-wise data split was enforced (Table 6).

**Table 6:**
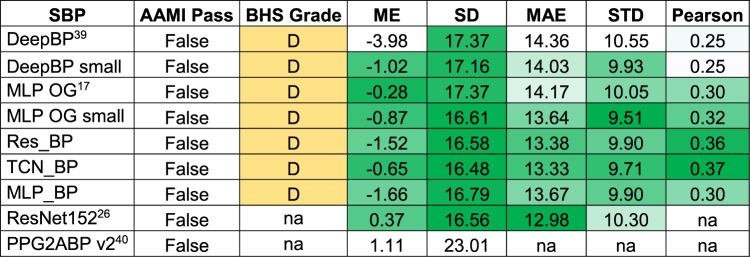
Subject-wise data split—SBP results.

In the second experiment, we repeated the training process, this time allowing segment data from each subject to appear in the training, validation, and test sets.

In this setting, we were able to reproduce the overall performance trends reported in prior works (Table 7), however, our Res_BP, TCN_BP, and MLP_BP models consistently underperformed the larger reference models. Assuming that data leakage contributes substantially to these results, a plausible contributing factor to the observed discrepancies is model capacity. DeepBP^37^ and the MLP model in^17^ contain approximately $2.1\times {10}^{6}$ and $5.9\times {10}^{7}$ total parameters (7.84 MB and 224 MB), respectively. A performance degradation was observed in the reduced-capacity variants DeepBP small and MLP OG small, which contain approximately $1.3\times {10}^{5}$ and $1.1\times {10}^{6}$ total parameters (0.50 MB and 4.03 MB), respectively. In contrast, our models without calibration processing comprise only $1.0\times {10}^{3}$ to $2.3\times {10}^{4}$ parameters (3.9 kB to 90.3 kB).

**Table 7:**
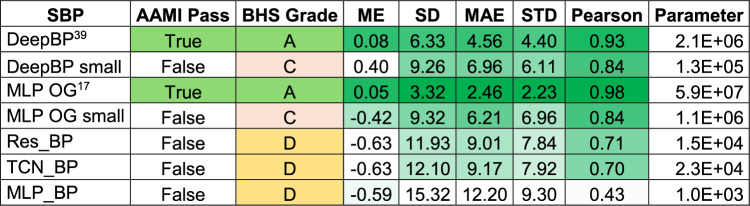
Data leakage split—SBP results.

Under data leakage conditions, the substantially larger DeepBP^37^ and MLP OG^17^ models likely benefited from their high representational capacity, enabling them to memorize subject-specific patterns rather than learn generalizable physiological relationships. This may also explain why MLP OG^17^, which is approximately 29 times larger than the second-largest model DeepBP^37^, achieved the best performance. Furthermore, progressively reducing the model capacity resulted in increased ME, SD, MAE, and STD values, suggesting that the smaller models approached their capacity limits and were therefore less capable of memorizing subject-specific information. This interpretation is further supported by the comparatively large effect sizes observed under data leakage conditions (*r* = 0.44 – 0.61), indicating substantial statistical differences between the large-capacity models and their reduced-capacity variants (Table 8).

**Table 8:** Statistics for pairwise comparison of the models with subject-wise data split and data leakage split—SBP results.

Pair	Outcome	Mean ΔMAE (mmHg)	95% CI	r
Subject wise split				
DeepBP^37^ vs MLP OG^17^	SBP	0.19	[0.10, 0.28]	0.03
TCN_BP vs MLP OG^17^	SBP	− 0.83	[− 0.90, − 0.76]	0.12
DeepBP^37^ vs DeepBP small	SBP	0.32	[0.26, 0.39]	0.05
MLP OG^17^ vs MLP OG small	SBP	0.52	[0.47, 0.58]	0.10
Data leakage				
DeepBP^37^ vs MLP OG^17^	SBP	2.1	[2.06, 2.15]	0.48
DeepBP^37^ vs DeepBP small	SBP	− 2.41	[− 2.46, − 2.35]	0.44
MLP OG^17^ vs MLP OG small	SBP	− 3.75	[− 3.82, − 3.69]	0.61

In contrast, when a strict subject-wise data split was applied, the larger models, DeepBP^37^ and MLP OG^17^, did not outperform their reduced-capacity counterparts, DeepBP small and MLP OG small. Although all pairwise comparisons remained statistically significant after correction for multiple comparisons (all *p* < 0.05), the observed differences in mean MAE and effect sizes were small (*r* = 0.03 – 0.12), indicating only minor practical differences between model variants despite statistical significance (Table 8). These findings suggest that, in the absence of data leakage, the larger capacities of the original models did not improve the performance of the reduced-capacity variants.

Interestingly, under the subject-wise data split condition, the largest performance difference was observed between the comparatively small TCN_BP model (90.3 kB) and the substantially larger MLP OG^17^ model (224 MB). Specifically, TCN_BP achieved a lower mean MAE difference (ΔMAE = − 0.83 mmHg) and the largest observed effect size in this setting (*r* = 0.12), indicating a slight performance advantage over MLP OG^17^. This suggests that the reduced capacity of the smaller model may have been beneficial, as it constrained the model to learn more generalizable representations rather than overfitting to subject-specific characteristics. This interpretation is further supported by slightly lower SD, MAE, and STD values, as well as higher Pearson correlation coefficients observed for the smaller models. However, given the small effect size, these findings should be interpreted with caution and do not imply that the smaller models consistently generalize better than their high-capacity counterparts.

A commonly used data augmentation strategy is to divide each segment into multiple shorter windows. While this approach can effectively increase the number of training samples, it requires careful handling to avoid data leakage. In datasets such as MIMIC-BP, where individual segments span 30 s, both the PPG waveform and the associated SBP and DBP values exhibit limited variability within a single segment, which could further amplify the memorization effect.

**Fig. 7:**
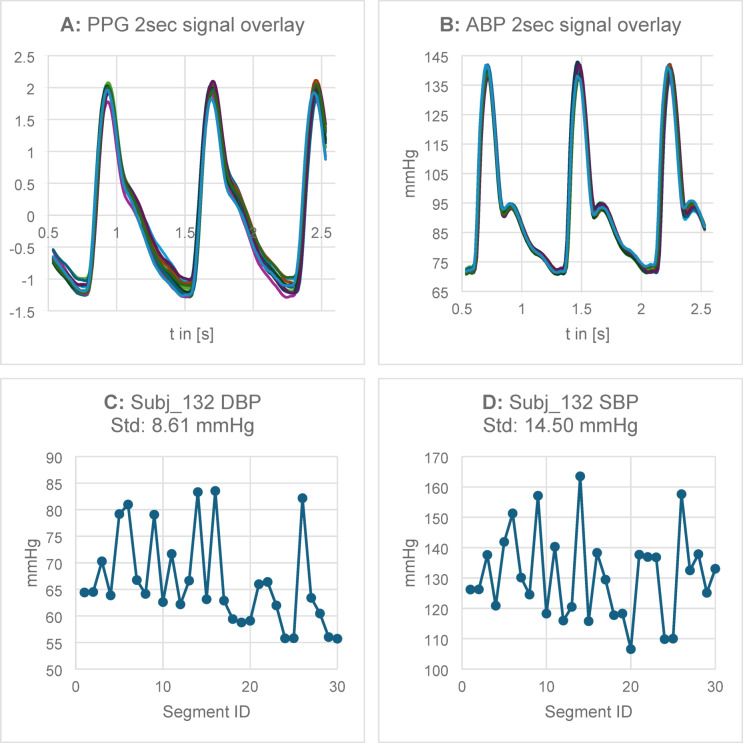
Overlayed 2 s PPG (**A**) and ABP (**B**) waves of one 30 s segment of subject 132. (**C**, **D**) displays the resulting DBP and SBP of all segments across one subject

For demonstration, we partitioned each 30 s MIMIC-BP segment from one subject into multiple non-overlapping 2 s windows and overlaid the corresponding PPG and ABP waveforms (see Fig. 7, windows A and B). SBP and DBP were computed as the maximum and minimum values within each 2 s window, respectively. The within-segment standard deviations were only 0.42 mmHg for DBP and 0.76 mmHg for SBP, indicating very limited blood pressure variability over the full 30 s interval and a high degree of similarity between the PPG and ABP signals.

Consequently, if multiple windows from the same segment are distributed across both training and test sets, a model may achieve artificially high accuracy by memorizing waveform–blood pressure pairs rather than learning physiologically meaningful relationships.

Nonetheless, the observed performance differences cannot be attributed solely to data leakage, as the present study also differs from^17,37^ in several other aspects, including the training and testing datasets, the applied filtering and preprocessing pipelines, the chosen training hyperparameters, and the choice of input data, such as signal length and scalar input features.

One could argue that the calibration approach used in this work also constitutes a form of data leakage. Here, the first three to nine measurements from separate segments were used for calibration. This stands in contrast to prior works, where data leakage is likely more pronounced: in those studies, all available samples were randomly shuffled into training and test sets, with samples from the same segment appearing in both^17,23,37^.

Using a few segments from the same subject—for example, reserving the first three to nine segments for calibration as well as using only one sample per segment—is substantially less prone to overfitting and memorization. As illustrated in Fig. 7C–D, the DBP and SBP values for a single subject vary noticeably across the 30 available segments (Segment DBP Std: 8.61 mmHg; Segment SBP Std: 14.50 mmHg). In this setting, the model cannot rely on simple memorization. Instead, it must learn patterns associated with blood pressure variation across time. When calibration data are available, the model additionally learns subject-specific offsets (e.g., associations between PAT and DBP, SBP), capturing how blood pressure deviates from an individualized baseline rather than memorizing absolute values.

### Comparison of the PPG data with the ABP data (Pulse_Vital)

To assess how signal fidelity and derived temporal features (e.g., PAT) affect prediction performance, we replaced the PPG waveform with the ABP waveform as the model input, using the Pulse_Vital set. To prevent data leakage and ensure subject-independent learning, the ABP signals were z-normalized at the individual subject level prior to model training.

The motivation for using ABP data lies in its superior representation of cardiovascular physiology. Unlike PPG, which provides an indirect optical measurement of peripheral blood volume changes and is highly sensitive to external factors such as sensor placement, contact pressure, skin properties, and motion artifacts, ABP directly measures intra-arterial pressure dynamics. As a result, the ABP waveform preserves critical morphological characteristics—including systolic upstroke, diastolic decay, and the dicrotic notch—that are closely linked to arterial compliance, vascular resistance, and wave reflection phenomena. These features are either distorted or partially lost in PPG recordings, limiting their physiological expressiveness for blood pressure estimation (compare PPG and ABP in Fig. 7A,B).

**Fig. 8:**
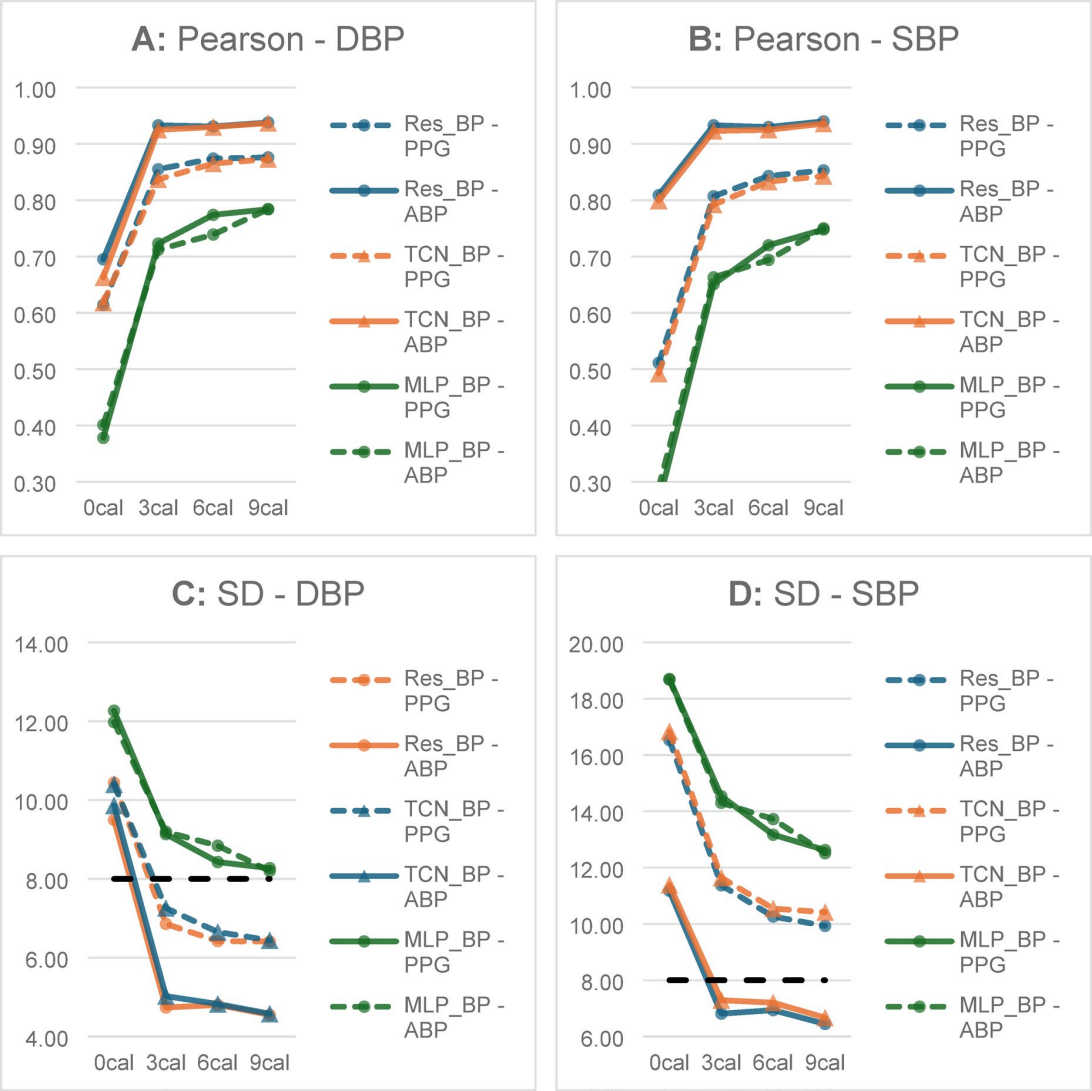
Model performances when the PPG or ABP waves are used as inputs. (**A**, **B**) displays the Pearson correlation. (**C**, **D**) SD according to AAMI

In our experiment, all models benefit from using ABP both as the input signal and for feature extraction (compare dashed PPG curves versus solid ABP curves in Fig. 8). The most pronounced improvements were observed in systolic blood pressure (SBP) prediction for models without calibration data, with Pearson correlation coefficients increasing by 58–62%. Notably, the TCN_BP and Res_BP configurations met the AAMI criteria for both DBP and SBP estimation when at least three calibration measurements were included. These models achieved Grade A performance for DBP and Grade A for SBP with Res_BP and Grade B for SBP with TCN_BP. In contrast, none of the PPG-based models satisfied the AAMI requirements for SBP estimation.

The statistical model comparison of Res_BP vs TCN_BP vs MLP_BP, after correction for multiple comparisons (all *p* < 0.05), revealed statistical significance, yet with small ΔMAE and effect sizes for TCN_BP vs Res_BP, indicating that these two models performed comparably, while Res_BP performed significantly better than MLP_BP, indicated by large ΔMAE and effect size. Exemplary for the condition with three calibrations:

$$ \begin{aligned} & {\mathrm{TCN}}\_{\text{BP vs Res}}\_{\mathrm{BP}}: \\ & {\mathrm{DBP}}:\Delta {\mathrm{MAE}} = 0.{4}\;{\mathrm{mmHg}},\;{95}\% \;{\text{CI }}\left[ {0.{28}, \, 0.{5}0} \right],r = \, 0.{19}; \\ & {\mathrm{SBP}}:\Delta {\mathrm{MAE}} = 0.{31}\;{\mathrm{mmHg}},\;{95}\% \;{\text{CI }}\left[ {0.{14}, \, 0.{48}} \right],r = \, 0.0{7}. \\ \end{aligned} $$

$$ \begin{aligned} & {\mathrm{Res}}\_{\text{BP vs MLP}}\_{\mathrm{BP}}: \\ & {\mathrm{DBP}}\Delta {\mathrm{MAE}} = - {3}.{36}\;{\mathrm{mmHg}},\;{95}\% \;{\text{CI }}\left[ { - {3}.{66}, \, - {3}.0{7}} \right],r = \, 0.{59}; \\ & {\mathrm{SBP}}:\Delta {\mathrm{MAE}} = - {5}.{67}\;{\mathrm{mmHg}},\;{95}\% \;{\text{CI }}\left[ { - {6}.{11}, \, - {5}.{23}} \right],r = \, 0.{66}. \\ \end{aligned} $$

A full overview of Model metrics and statistics across no, three, six, and nine calibrations for DBP and SBP is provided in the Supplementary Data.

Interestingly, a slight increase in SD was observed when the number of calibration measurements increased from three to six in the ABP setting (Fig. 8). Although the differences were small overall, this finding suggests that additional calibration measurements did not lead to a strictly monotonic improvement in performance in this dataset. Notably, the SD obtained with nine calibration measurements was again slightly lower than that observed with three calibrations, indicating a non-linear relationship between the number of calibration samples and model performance. One possible explanation is that subject-specific adjustment may already be adequately captured using a small number of calibration samples, while intermediate increases in calibration measurements could introduce additional variability related to physiological fluctuations or measurement noise. With a larger number of calibration samples, however, the effect of such variability may be partially compensated by averaging across more measurements. However, these interpretations remain speculative, as they were not explicitly tested in the present study. In addition, the observed pattern may be sensitive to the random seed used (seed 42). Further robustness analysis using multiple random seeds and repeated training runs would be necessary to assess the stability of this trend.

Finally, Res_BP and TCN_BP demonstrated larger performance improvements when using ABP waveform signals. MLP_BP model, which relied solely on scalar features such as PAT derived from ECG-to-PPG or -ABP, showed only minimal or no improvement. This suggests that although MLP_BP underperforms overall compared with Res_BP and TCN_BP, the scalar features used in this study (e.g., PAT) are less sensitive to temporal shifts when extracted from PPG or ABP signals.

**Fig. 9:**
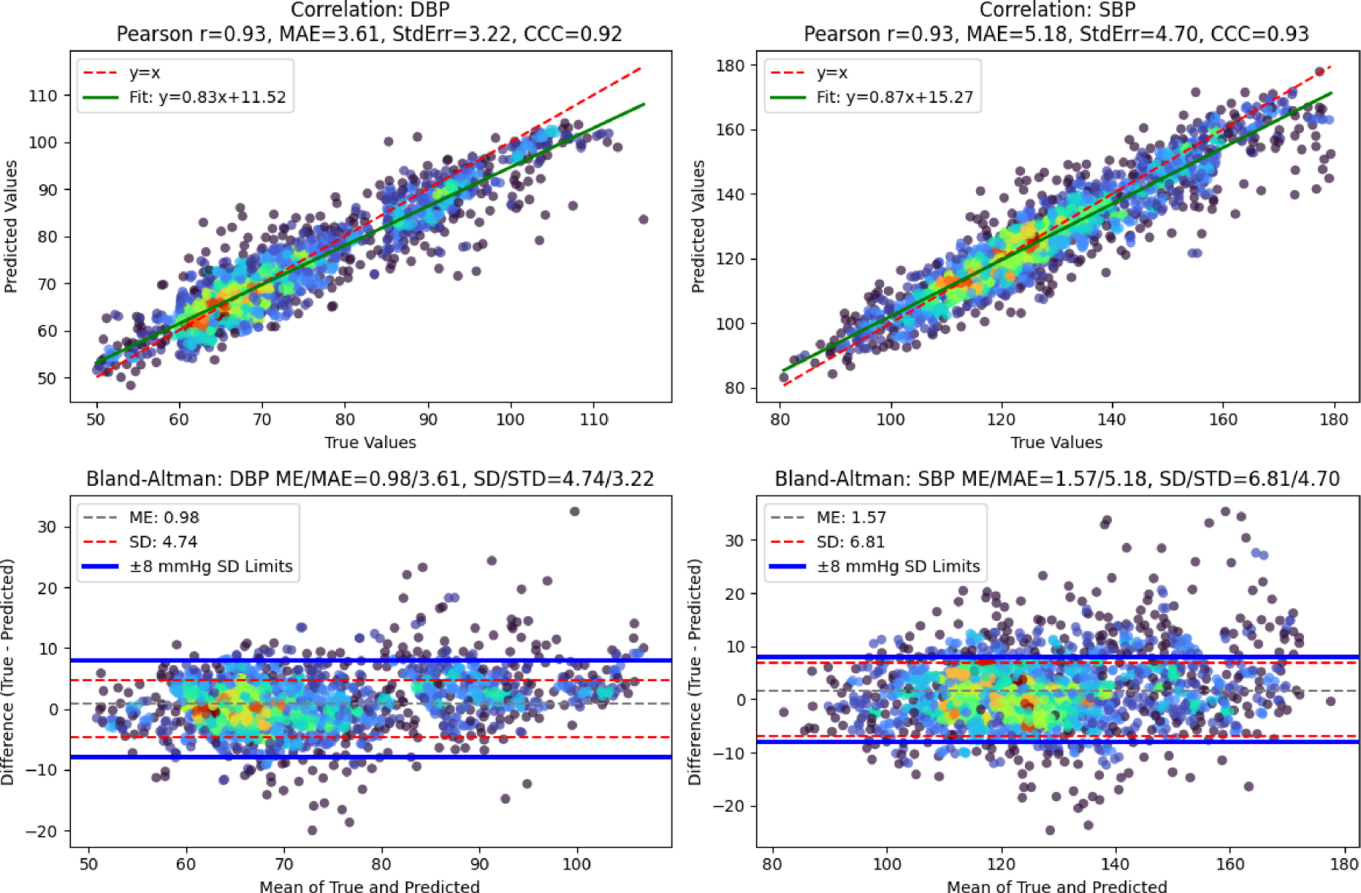
Correlation and Bland–Altman plot of DBP and SBP for the best performing model, Res_BP with three calibration measurements. The rainbow color coding shows the amount of the data, with red indicating high amount of data and blue indicating low amount of data

The predictive performance of the Res_BP model, calibrated with three measurements, was assessed for diastolic blood pressure (DBP) and systolic blood pressure (SBP) via correlation and Bland–Altman analyses (Fig. 9). Density-colored scatterplots indicate that regions with higher sample density, corresponding to more frequently observed BP values, are predicted more accurately by the model.

**Fig. 10:**
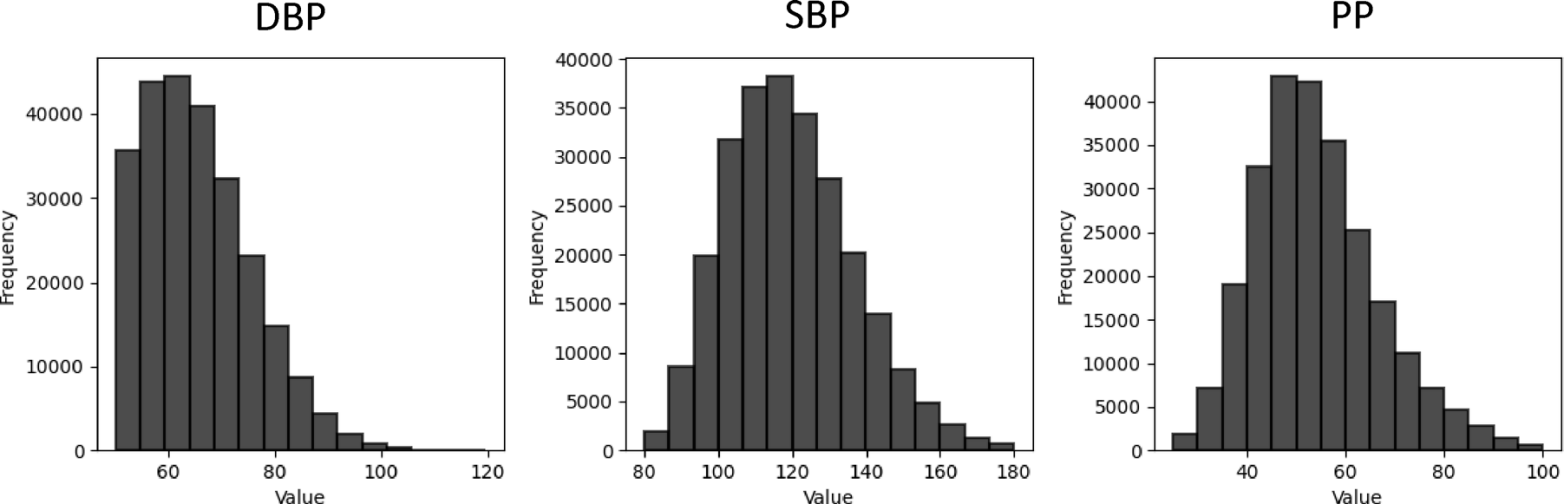
Histogram of DBP, SBP and PP in the Pulse_Vital Train Set

Especially for SBP, higher values tend to exhibit larger prediction errors, with the model generally underestimating true SBP. This pattern has also been observed in previous studies, as summarized by^7^, who attributed it to an imbalance phenomenon in BP datasets, in which skewed distributions bias predictions toward central BP values. In Fig. 10, for example, the number of samples with SBP > 160 mmHg was substantially lower than that in lower ranges. No strategies to correct this imbalance were applied in this work.

Overall, the findings demonstrate that the blood-flow representation captured by PPG does not adequately encode the pressure-related information required for reliable SBP prediction in the Res_BP and TCN_BP models, which jointly process waveform and scalar features. The larger performance improvements observed for SBP indicate that systolic pressure estimation is more sensitive to signal fidelity than DBP estimation. As illustrated in Fig. 7 (A vs B), the PPG waveform fails to reproduce key pressure wave characteristics, especially visible with the missing dicrotic notch.

In contrast, DBP could be estimated with acceptable accuracy from PPG signals when at least three calibration measurements were available. However, even the ABP-based models required a minimum of three calibration measurements to meet the AAMI performance criterion for DBP estimation.

## Conclusion

### Influence model architecture

In this study, we systematically evaluated neural network architectures for cuffless blood pressure estimation and demonstrated that performance gains arise primarily from two factors: multimodal feature fusion and dataset quality. Extending a scalar-processing MLP with a dedicated waveform-processing branch substantially improved prediction accuracy compared with scalar-only models. Notably, the specific waveform architecture (ResNet versus TCN) had less impact than the overarching strategy of integrating physiologically interpretable scalar descriptors (such as PAT and HRV) with learned waveform representations. These findings highlight the importance of combining complementary feature domains. Including the PP auxiliary head did not affect prediction accuracy within the current architecture, where DBP, SBP and PP are predicted from a shared latent representation.

### Dataset selection

Dataset selection exerted an even greater influence on performance. Transitioning from MIMIC-based datasets to the temporally synchronized Pulse_Vital dataset (based on VitalDB) improved SBP prediction accuracy, particularly when ABP waveforms were used. This improvement likely reflects reliable inter-channel synchronization, which enables more precise PAT estimation. In contrast, time-varying inter-signal delays introduce irreducible noise that limits model performance. Differences in patient populations—ICU versus surgical cohorts—may further contribute to the observed performance gap, as hemodynamic instability and vasoactive medication exposure alter vascular tone and pulse wave propagation.

### Subject-specific calibration and waveform representation

Prediction accuracy improved most strongly when the waveform-and-scalar-feature-based models (Res_BP and TCN_BP) were trained directly on ABP waveforms and when subject-specific calibration was applied. Unlike PPG, ABP directly reflects arterial pressure dynamics, allowing the model to capture waveform characteristics critical for SBP and DBP estimation. Calibration further addressed the inherently individual relationship between PAT and blood pressure, enabling personalized modeling of baseline offsets and temporal variations. In contrast, the scalar feature-based model (MLP_BP) showed little sensitivity to whether features were derived from PPG or ABP signals, with only minor differences in prediction accuracy.

### Limitations and outlook

In this work, calibration was based on the first three to nine reference measurements per subject. Future studies should examine how calibration strategy, timing, and physiological diversity influence robustness. Acquiring calibration data under varied conditions—such as sleep, physical activity, and rest—may span a broader range of blood pressure and PAT values and thereby enable more generalizable personalization.

Due to computational constraints, all experiments were conducted using a fixed random seed to ensure reproducibility. While this approach provides consistency in reported results, a comprehensive evaluation across multiple random seeds was not performed. Such an analysis is recommended in future studies to assess the stability and variability of model training.

Beyond calibration and model design, advances in measurement technology are likely to be equally important. Our results show that richer and more physiological waveform inputs substantially improve prediction accuracy, underscoring the need for sensors and acquisition methods that more closely approximate arterial pressure dynamics.

Finally, all datasets analyzed here were derived from ICU or surgical populations. Whether these findings generalize to healthy individuals in ambulatory settings remains uncertain, particularly given the influence of vasoactive medications on vascular tone and PAT. Moreover, exercise-induced high SBP ranges (e.g., ≥ 210 mmHg in males) are not represented in commonly used datasets derived from MIMIC and VitalDB. The development of datasets that include healthy subjects and a broader range of blood pressure values will be essential to improve external validity and real-world applicability.

## Supplementary Information

Below is the link to the electronic supplementary material.

Supplementary Material 1

## Data Availability

In this study, publicly available datasets were used for analysis and training. The used datasets are available under the following links:—MIMIC-BP: 10.7910/DVN/DBM1NF-PulseDB: https://github.com/pulselabteam/PulseDB Our Models are available on GitHub: —https://github.com/ga38sit/bp_prediction_withCal.git

## References

[1] Fuchs, F. D. & Whelton, P. K. High blood pressure and cardiovascular disease. *Hypertension* **75**(2), 285–292. 10.1161/HYPERTENSIONAHA.119.14240 (2020). 31865786 10.1161/HYPERTENSIONAHA.119.14240 PMC10243231

[2] World Health Organization, “Global report on hypertension: the race against a silent killer.” Accessed: Nov. 07, 2025. [Online]. Available: https://www.who.int/publications/i/item/9789240081062

[3] Zhao, L. et al. Emerging sensing and modeling technologies for wearable and cuffless blood pressure monitoring. *NPJ Digit. Med.* **6**(1), 93. 10.1038/s41746-023-00835-6 (2023). 37217650 10.1038/s41746-023-00835-6 PMC10203315

[4] Pilz, N. et al. Cuff-based blood pressure measurement: Challenges and solutions. *Blood Press.*10.1080/08037051.2024.2402368 (2024). 39291896 10.1080/08037051.2024.2402368

[5] Müller, P., Lechner, K., Halle, M. & Braun-Dullaeus, R. Physical activity and arterial hypertension. *Dtsch. Z. Sportmed.* **74**(3), 74–79. 10.5960/dzsm.2023.560 (2023).

[6] Pressler, A., Jähnig, A., Halle, M. & Haller, B. Blood pressure response to maximal dynamic exercise testing in an athletic population. *J. Hypertens.* **36**(9), 1803–1809. 10.1097/HJH.0000000000001791 (2018). 29794559 10.1097/HJH.0000000000001791

[7] Qin, K., Huang, W., Zhang, T. & Tang, S. Machine learning and deep learning for blood pressure prediction: A methodological review from multiple perspectives. *Artif. Intell. Rev.* **56**(8), 8095–8196. 10.1007/s10462-022-10353-8 (2023).

[8] Elgendi, M., Liang, Y. & Ward, R. Toward generating more diagnostic features from photoplethysmogram waveforms. *Diseases* **6**(1), 20. 10.3390/diseases6010020 (2018). 29534495 10.3390/diseases6010020 PMC5871966

[9] Sarkar, S. & Ghosh, A. Schrödinger spectrum based continuous cuff-less blood pressure estimation using clinically relevant features from PPG signal and its second derivative. *Comput. Biol. Med.* **166**, 107558. 10.1016/j.compbiomed.2023.107558 (2023). 37806054 10.1016/j.compbiomed.2023.107558

[10] Li, P. & Laleg-Kirati, T.-M. Central blood pressure estimation from distal PPG measurement using semiclassical signal analysis features. *IEEE Access* **9**, 44963–44973. 10.1109/ACCESS.2021.3065576 (2021).

[11] Finnegan, E. et al. Pulse arrival time as a surrogate of blood pressure. *Sci. Rep.* **11**(1), 22767. 10.1038/s41598-021-01358-4 (2021). 34815419 10.1038/s41598-021-01358-4 PMC8611024

[12] Esmaili, A., Kachuee, M. & Shabany, M. Nonlinear cuffless blood pressure estimation of healthy subjects using pulse transit time and arrival time. *IEEE Trans. Instrum. Meas.* **66**(12), 3299–3308. 10.1109/TIM.2017.2745081 (2017).

[13] Goldberger, A. L. et al. PhysioBank, PhysioToolkit, and PhysioNet. *Circulation* **101**, 23. 10.1161/01.CIR.101.23.e215 (2000). 10.1161/01.cir.101.23.e215 10851218

[14] Wang, W., Mohseni, P., Kilgore, K. L. & Najafizadeh, L. Cuff-less blood pressure estimation from photoplethysmography via visibility graph and transfer learning. *IEEE J. Biomed. Health Inform.* **26**(5), 2075–2085. 10.1109/JBHI.2021.3128383 (2022). 34784289 10.1109/JBHI.2021.3128383

[15] Subramanian, S., Mishra, S., Patil, S., Kolekar, M. H. & Ortiz-Rodriguez, F. Integrating transfer learning with scalogram analysis for blood pressure estimation from PPG signals. *Sci. Rep.* **15**(1), 39647. 10.1038/s41598-025-23350-y (2025). 41224941 10.1038/s41598-025-23350-y PMC12612122

[16] Esmaelpoor, J., Moradi, M. H. & Kadkhodamohammadi, A. A multistage deep neural network model for blood pressure estimation using photoplethysmogram signals. *Comput. Biol. Med.* **120**, 103719. 10.1016/j.compbiomed.2020.103719 (2020). 32421641 10.1016/j.compbiomed.2020.103719

[17] Hsu, Y.-C., Li, Y.-H., Chang, C.-C. & Harfiya, L. N. Generalized deep neural network model for cuffless blood pressure estimation with photoplethysmogram signal only. *Sensors* **20**(19), 5668. 10.3390/s20195668 (2020). 33020401 10.3390/s20195668 PMC7582614

[18] Tian, Z., Liu, A., Zhu, G. & Chen, X. A paralleled CNN and transformer network for PPG-based cuff-less blood pressure estimation. *Biomed. Signal Process. Control* **99**, 106741. 10.1016/j.bspc.2024.106741 (2025).

[19] Darbhasayanam, S. H., Noorjahan, H. A., Mohammad, A. & Komalla, A. R. Photoplethysmogram (PPG)-based blood pressure estimation using vision transformer networks: A deep learning approach. In *2024 3rd International Conference on Artificial Intelligence For Internet of Things (AIIoT) *1–6 (IEEE 2024). 10.1109/AIIoT58432.2024.10574539

[20] El-Hajj, C. & Kyriacou, P. Deep learning models for cuffless blood pressure monitoring from PPG signals using attention mechanism. *Biomed. Signal Process. Control* **65**, 102301. 10.1016/j.bspc.2020.102301 (2021).

[21] Stergiou, G. S. et al. A universal standard for the validation of blood pressure measuring devices. *Hypertension* **71**(3), 368–374. 10.1161/HYPERTENSIONAHA.117.10237 (2018). 29386350 10.1161/HYPERTENSIONAHA.117.10237

[22] O’Brien, E., Waeber, B., Parati, G., Staessen, J. & Myers, M. G. Blood pressure measuring devices: recommendations of the European Society of Hypertension. *BMJ* **322**(7285), 531–536. 10.1136/bmj.322.7285.531 (2001). 11230071 10.1136/bmj.322.7285.531 PMC1119736

[23] Yu, M., Huang, Z., Zhu, Y., Zhou, P. & Zhu, J. Attention-based residual improved U-Net model for continuous blood pressure monitoring by using photoplethysmography signal. *Biomed. Signal Process. Control* **75**, 103581. 10.1016/j.bspc.2022.103581 (2022).

[24] Mehta, S., Kwatra, N., Jain, M. & McDuff, D. Examining the challenges of blood pressure estimation via photoplethysmogram. *Sci. Rep.* **14**(1), 18318. 10.1038/s41598-024-68862-1 (2024). 39112533 10.1038/s41598-024-68862-1 PMC11306225

[25] Schrumpf, F., Frenzel, P., Aust, C., Osterhoff, G. & Fuchs, M. Assessment of non-invasive blood pressure prediction from PPG and rPPG signals using deep learning. *Sensors* **21**(18), 6022. 10.3390/s21186022 (2021). 34577227 10.3390/s21186022 PMC8472879

[26] Sanches, I. et al. MIMIC-BP: A curated dataset for blood pressure estimation. *Sci. Data* **11**(1), 1233. 10.1038/s41597-024-04041-1 (2024). 39548096 10.1038/s41597-024-04041-1 PMC11568151

[27] Wang, W., Mohseni, P., Kilgore, K. L. & Najafizadeh, L. PulseDB: A large, cleaned dataset based on MIMIC-III and VitalDB for benchmarking cuff-less blood pressure estimation methods. *Front. Digit. Health*10.3389/fdgth.2022.1090854 (2023). 36844249 10.3389/fdgth.2022.1090854 PMC9944565

[28] Moody, B., Moody, G., Villaroel, M., Clifford, G. D. & Silva, I. MIMIC-III Waveform Database Matched Subset (version 1.0), 2020. Accessed: Nov. 21, 2025. [Online]. Available: 10.13026/c2294b

[29] Moody, B., Moody, G., Villarroel, M., Clifford, G. D. & Silva, I. MIMIC-III Waveform Database (version 1.0), *PhysioNet*, 2020, Accessed: Jan. 12, 2026. [Online]. Available: 10.13026/c2607m

[30] “VitalDB.” Accessed: Jan. 12, 2026. [Online]. Available: https://vitaldb.net/dataset/

[31] Mukkamala, R. et al. Toward ubiquitous blood pressure monitoring via pulse transit time: Theory and practice.. *IEEE Trans. Biomed. Eng.* **62**(8), 1879–1901. 10.1109/TBME.2015.2441951 (1879). 10.1109/TBME.2015.2441951 PMC4515215 26057530

[32] O’Brien, E. et al. The British Hypertension Society protocol for the evaluation of automated and semi-automated blood pressure measuring devices with special reference to ambulatory systems. *J. Hypertens.* **8**(7), 607–619. 10.1097/00004872-199007000-00004 (1990). 2168451 10.1097/00004872-199007000-00004

[33] Liang, Y. et al. How effective is pulse arrival time for evaluating blood pressure? Challenges and recommendations from a study using the MIMIC database.. *J. Clin. Med.*10.3390/jcm8030337 (2019). 30862031 10.3390/jcm8030337 PMC6462898

[34] Escobar-Restrepo, B., Torres-Villa, R. & Kyriacou, P. A. Evaluation of the linear relationship between pulse arrival time and blood pressure in ICU patients: Potential and limitations. *Front. Physiol.*10.3389/fphys.2018.01848 (2018). 30622482 10.3389/fphys.2018.01848 PMC6308183

[35] Lee, J., Yang, S., Lee, S. & Kim, H. C. Analysis of pulse arrival time as an indicator of blood pressure in a large surgical biosignal database: Recommendations for developing ubiquitous blood pressure monitoring methods. *J. Clin. Med.*10.3390/jcm8111773 (2019). 31653002 10.3390/jcm8111773 PMC6912522

[36] Wunderle, M. et al. Continuous measurement of pulse arrival time for identification of blood pressure changes in patients undergoing hemodialysis—A feasibility study. *Clin. Kidney J.*10.1093/ckj/sfaf255 (2025). 41215784 10.1093/ckj/sfaf255 PMC12596194

[37] Yan, C. et al., Novel deep convolutional neural network for cuff-less blood pressure measurement using ECG and PPG signals. In *2019 41st Annual International Conference of the IEEE Engineering in Medicine and Biology Society (EMBC)* 1917–1920 (IEEE, 2019). 10.1109/EMBC.2019.8857108 10.1109/EMBC.2019.8857108 31946273

[38] Mehta, S., Kwatra, N., Jain, M. & McDuff, D. Examining the challenges of blood pressure estimation via photoplethysmogram. *Sci. Rep.* **14**(1), 18318. 10.1038/s41598-024-68862-1 (2024). 39112533 10.1038/s41598-024-68862-1 PMC11306225

