# Supplementary material for: The role of dataset integrity, calibration and signal quality in neural network-based blood pressure estimation
Source: Sci Rep. 2026 Aug 15;16:25524. doi: 10.1038/s41598-026-66882-7 (PMC13477474; doi:10.1038/s41598-026-66882-7)
Supplement: Supplementary file 1 [file 41598_2026_66882_MOESM1_ESM.pdf]

Models on GitHub: https://github.com/ga38sit/bp_prediction_withCal.git

Table 1: Statistical comparison between the models trained with Pulse_Vital and normalized PPG wave as signal input

0cal					
**Pair**	**Outcome**	**Mean ΔMAE (mmHg)**	**95% CI**	**p (corr)**	**r**
TCN_BP vs Res_BP	DBP	0.16	[0.03, 0.29]	0.040	0.07
TCN_BP vs MLP_BP	DBP	-0.97	[-1.25, -0.70]	< 0.001	0.16
Res_BP vs MLP_BP	DBP	-1.14	[-1.41, -0.86]	< 0.001	0.19
TCN_BP vs Res_BP	SBP	0.33	[0.10, 0.55]	0.040	0.07
TCN_BP vs MLP_BP	SBP	-1.07	[-1.45, -0.70]	< 0.001	0.14
Res_BP vs MLP_BP	SBP	-1.4	[-1.79, -1.02]	< 0.001	0.18

3cal					
**Pair**	**Outcome**	**Mean ΔMAE (mmHg)**	**95% CI (mmHg)**	**p (corr)**	**r**
TCN_BP vs Res_BP	DBP	0.06	[-0.06, 0.18]	0.874	0.01
TCN_BP vs MLP_BP	DBP	-1.7	[-1.90, -1.49]	< 0.001	0.45
Res_BP vs MLP_BP	DBP	-1.76	[-1.99, -1.55]	< 0.001	0.42
TCN_BP vs Res_BP	SBP	0.08	[-0.11, 0.26]	0.808	0.02
TCN_BP vs MLP_BP	SBP	-2.59	[-2.96, -2.23]	< 0.001	0.38
Res_BP vs MLP_BP	SBP	-2.66	[-3.05, -2.28]	< 0.001	0.36

6cal					
**Pair**	**Outcome**	**Mean ΔMAE (mmHg)**	**95% CI**	**p (corr)**	**r**
TCN vs ResNet	DBP	0.3	[0.20, 0.39]	< 0.001	0.14
TCN vs MLP	DBP	-1.56	[-1.77, -1.35]	< 0.001	0.38
ResNet vs MLP	DBP	-1.86	[-2.09, -1.64]	< 0.001	0.42
TCN vs ResNet	SBP	0.42	[0.26, 0.58]	< 0.001	0.15
TCN vs MLP	SBP	-1.97	[-2.31, -1.64]	< 0.001	0.29
ResNet vs MLP	SBP	-2.39	[-2.73, -2.04]	< 0.001	0.35

9cal					
**Pair**	**Outcome**	**Mean ΔMAE (mmHg)**	**95% CI**	**p (corr)**	**r**
TCN_BP vs Res_BP	DBP	0.052	[-0.046, 0.150]	0.351	0.027
TCN_BP vs MLP_BP	DBP	-1.48	[-1.694, -1.269]	< 0.001	0.349
Res_BP vs MLP_BP	DBP	-1.532	[-1.734, -1.330]	< 0.001	0.384
TCN_BP vs Res_BP	SBP	0.269	[0.098, 0.446]	0.005	0.088
TCN_BP vs MLP_BP	SBP	-1.901	[-2.253, -1.556]	< 0.001	0.272
Res_BP vs MLP_BP	SBP	-2.17	[-2.491, -1.834]	< 0.001	0.349

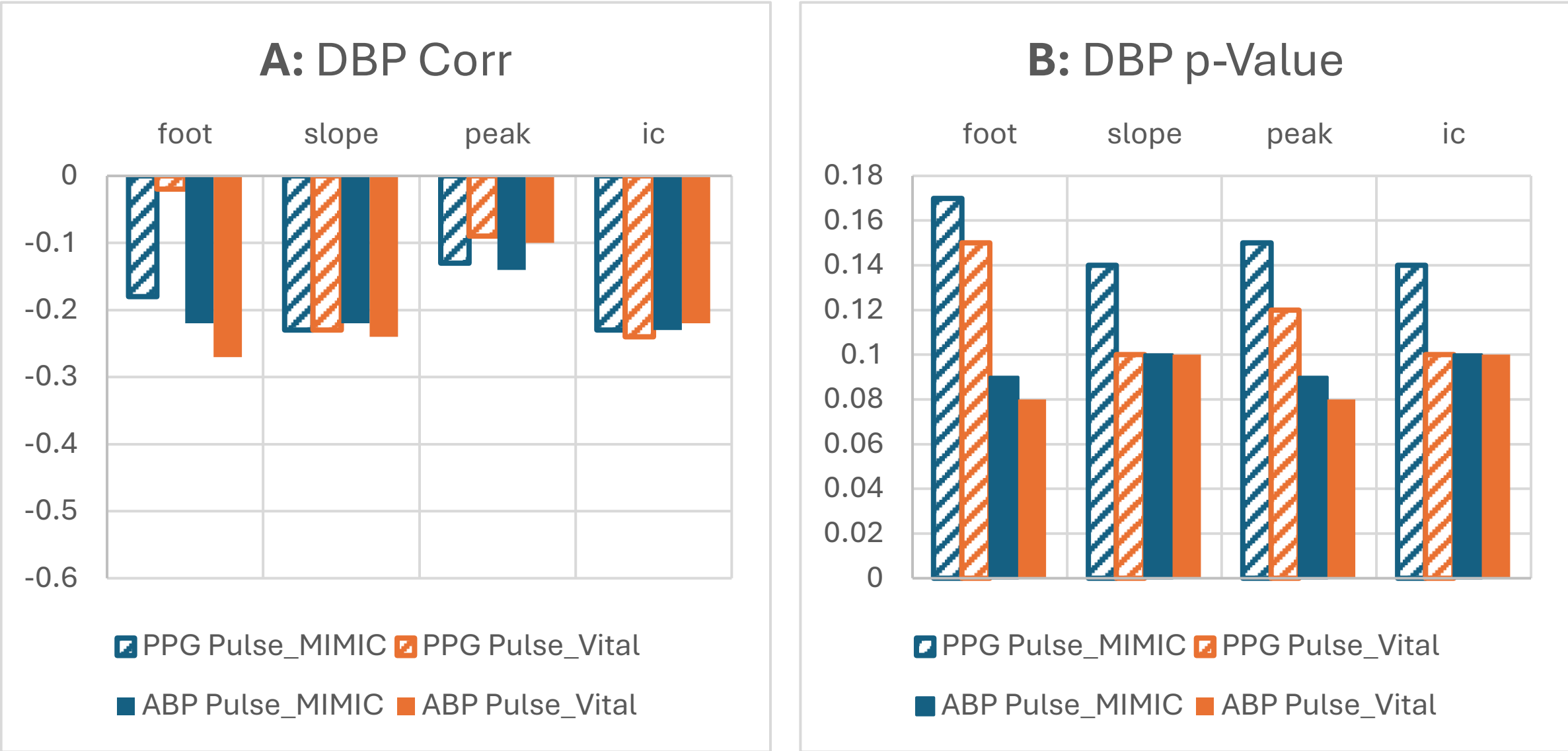

Figure 1: Pearson correlation coefficients for DBP (A), and corresponding p-values for DBP (B), evaluated on the Pulse_MIMIC and Pulse_Vital datasets via PPG or ABP signals

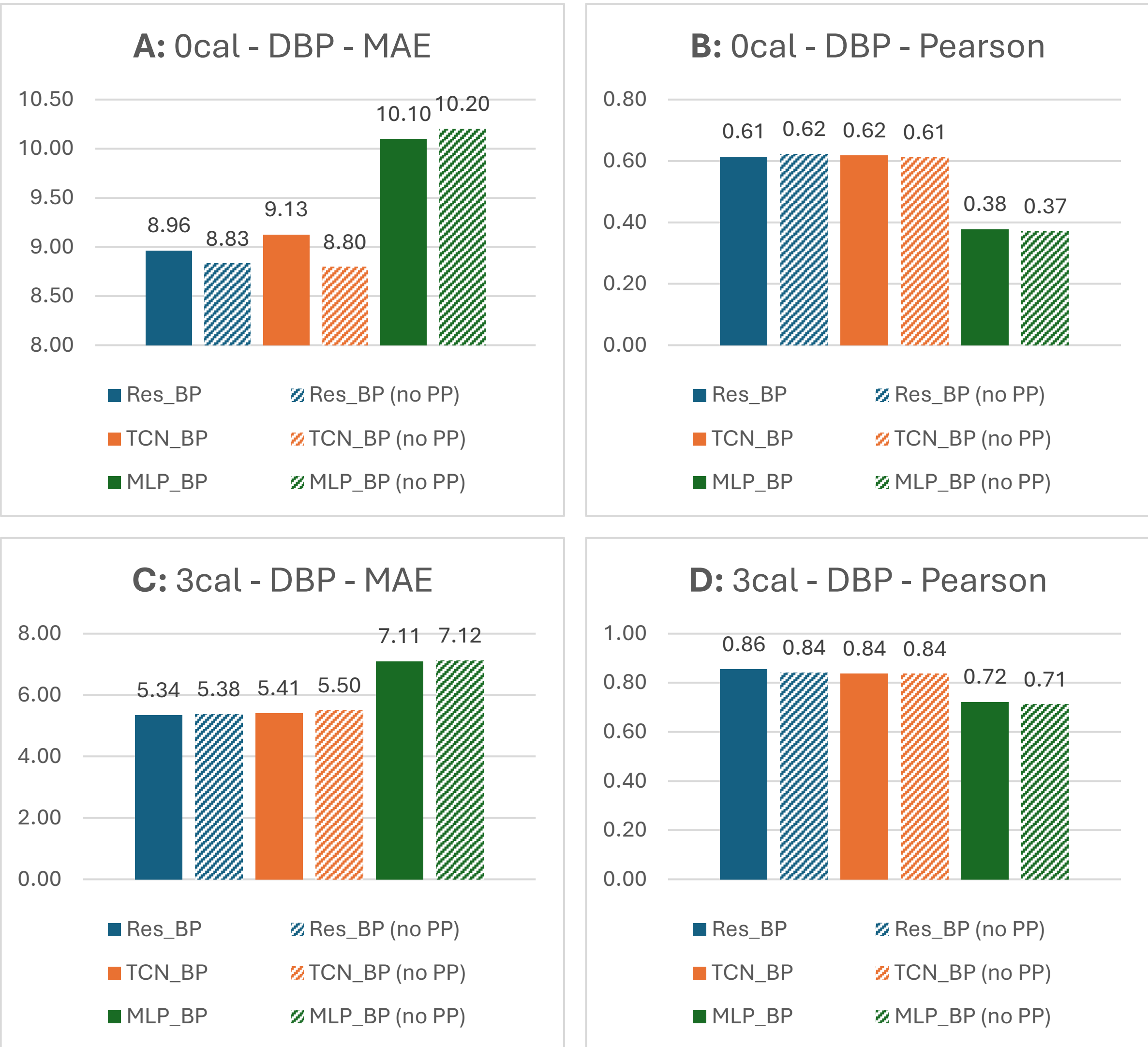

Figure 2: Comparison of Model Performances for DBP Prediction with and without (no PP) auxiliary PP head

Table 2: Comparison of the DBP Metrics of models trained with Pulse_Vital with normalized ABP wave as signal input

Pulse_Vital	DBP	AAMI Pass	BHS Grade	ME	SD	MAE	STD	Pearson
Res_BP	0cal	False	D	-4.85	9.50	8.27	6.73	0.70
	3cal	True	A	-0.98	4.74	3.61	3.22	0.93
	6cal	True	A	-0.74	4.80	3.54	3.33	0.93
	9cal	True	A	-1.14	4.55	3.43	3.20	0.94
TCN_BP	0cal	False	D	-3.68	9.86	8.14	6.67	0.66
	3cal	True	A	-1.74	5.02	4.00	3.49	0.93
	6cal	True	A	-0.80	4.83	3.59	3.32	0.93
	9cal	True	A	-1.16	4.58	3.44	3.24	0.94
MLP_BP	0cal	False	D	-3.42	11.98	9.51	8.04	0.40
	3cal	False	C	-3.47	9.19	6.97	6.92	0.71
	6cal	False	B	-2.93	8.84	6.64	6.53	0.74
	9cal	False	B	-2.68	8.20	6.26	5.93	0.78

Table 3: Comparison of the SBP Metrics of models trained with Pulse_Vital with normalized ABP wave as signal input

Pulse_Vital	SBP	AAMI Pass	BHS Grade	ME	SD	MAE	STD	Pearson
Res_BP	0cal	False	D	-3.76	11.18	9.04	7.57	0.81
	3cal	True	A	-1.57	6.81	5.18	4.70	0.93
	6cal	True	A	-1.25	6.94	5.20	4.76	0.93
	9cal	True	A	-1.12	6.45	4.83	4.41	0.94
TCN_BP	0cal	False	D	-4.02	11.38	9.28	7.72	0.80
	3cal	True	B	-1.52	7.29	5.49	5.03	0.92
	6cal	True	B	-0.75	7.20	5.28	4.96	0.93
	9cal	True	A	-1.14	6.67	4.91	4.65	0.94
MLP_BP	0cal	False	D	1.17	18.68	15.34	10.71	0.29
	3cal	False	D	-4.26	14.30	10.85	10.24	0.66
	6cal	False	D	-3.84	13.72	10.41	9.73	0.69
	9cal	False	D	-2.66	12.51	9.50	8.56	0.75

Table 4: Statistical comparison between the models trained with Pulse_Vital and normalized ABP wave as signal input

0cal					
**Pair**	**Outcome**	**Mean ΔMAE (mmHg)**	**95% CI**	**p (corr)**	**r**
TCN_BP vs Res_BP	DBP	-0.13	[-0.33, 0.07]	0.091	0.06
TCN_BP vs MLP_BP	DBP	-1.98	[-2.34, -1.60]	< 0.001	0.23
Res_BP vs MLP_BP	DBP	-1.85	[-2.25, -1.46]	< 0.001	0.19
TCN_BP vs Res_BP	SBP	0.24	[0.01, 0.50]	0.244	0.03
TCN_BP vs MLP_BP	SBP	-5.67	[-6.21, -5.12]	< 0.001	0.51
Res_BP vs MLP_BP	SBP	-5.91	[-6.48, -5.37]	< 0.001	0.53

3cal					
**Pair**	**Outcome**	**Mean ΔMAE (mmHg)**	**95% CI (mmHg)**	**p (corr)**	**r**
TCN_BP vs Res_BP	DBP	0.40	[0.28, 0.50]	< 0.001	0.19
TCN_BP vs MLP_BP	DBP	-2.97	[-3.24, -2.69]	< 0.001	0.52
Res_BP vs MLP_BP	DBP	-3.36	[-3.66, -3.07]	< 0.001	0.59
TCN_BP vs Res_BP	SBP	0.31	[0.14, 0.48]	0.019	0.07
TCN_BP vs MLP_BP	SBP	-5.36	[-5.79, -4.94]	< 0.001	0.62
Res_BP vs MLP_BP	SBP	-5.67	[-6.11, -5.23]	< 0.001	0.66

6cal					
**Pair**	**Outcome**	**Mean ΔMAE (mmHg)**	**95% CI**	**p (corr)**	**r**
TCN_BP vs Res_BP	DBP	0.05	[-0.06, 0.15]	1.000	0.01
TCN_BP vs MLP_BP	DBP	-3.05	[-3.32, -2.77]	< 0.001	0.57
Res_BP vs MLP_BP	DBP	-3.1	[-3.37, -2.82]	< 0.001	0.59
TCN_BP vs Res_BP	SBP	0.07	[-0.09, 0.23]	1.000	0.01
TCN_BP vs MLP_BP	SBP	-5.13	[-5.56, -4.71]	< 0.001	0.60
Res_BP vs MLP_BP	SBP	-5.2	[-5.63, -4.78]	< 0.001	0.63

9cal					
**Pair**	**Outcome**	**Mean ΔMAE (mmHg)**	**95% CI**	**p (corr)**	**r**
TCN_BP vs Res_BP	DBP	0.01	[-0.08, 0.10]	1.000	0.01
TCN_BP vs MLP_BP	DBP	-2.83	[-3.08, -2.57]	< 0.001	0.57
Res_BP vs MLP_BP	DBP	-2.84	[-3.09, -2.59]	< 0.001	0.57
TCN_BP vs Res_BP	SBP	0.09	[-0.07, 0.24]	1.000	0.00
TCN_BP vs MLP_BP	SBP	-4.58	[-4.96, -4.20]	< 0.001	0.60
Res_BP vs MLP_BP	SBP	-4.67	[-5.05, -4.29]	< 0.001	0.61
